# Clinical Significance of Serum Biomarkers in Stage IV Non-Small-Cell Lung Cancer Treated with PD-1 Inhibitors: LIPI Score, NLR, dNLR, LMR, and PAB

**DOI:** 10.1155/2022/7137357

**Published:** 2022-07-30

**Authors:** Jia Chen, Sheng Wei, Tianye Zhao, Xunlei Zhang, Yilang Wang, Xiaodong Zhang

**Affiliations:** ^1^Department of Medical Oncology, The Affiliated Tumor Hospital of Nantong University & Nantong Tumor Hospital, Nantong, China; ^2^Nantong University, Nantong, China

## Abstract

**Background:**

To assess the prognostic value of pretreatment serum biomarkers in stage IV non-small-cell lung cancer (NSCLC) patients treated with PD-1 (programmed cell death protein 1) inhibitors and their value as a predictor of benefit.

**Methods:**

We performed a retrospective study including patients with stage IV NSCLC who were treated with anti-PD-1 drugs in first or advanced lines of therapy in the Affiliated Tumor Hospital of Nantong University. Serum biomarkers such as NLR, dNLR, LMR, PAB, ALB, and LIPI scores were calculated and analyzed in detail.

**Results:**

A total of 85 patients with stage IV NSCLC treated with PD-1 inhibitors in the first or advanced lines of therapy were included in this subject. According to the tumor response of PD-1-based treatment, ORR was 42.4% (36/85) and DCR was 68.2% (58/85). The median OS and PFS were 20.0 months and 7.0 months, respectively. The ROC curves showed that the serum biomarkers of NLR, dNLR, LDH, LMR, PAB, and ALB were significantly associated with overall survival and helped to determine the cut-off value. The multivariate Cox proportional hazard analyses for stage IV NSCLC patients treated with PD-1 inhibitors indicated that dNLR (*P* < 0.001) and ALB (*P* = 0.033) were independent prognostic indicators of PFS, while liver metastasis (*P* = 0.01), NLR (*P* = 0.01), dNLR (*P* = 0.001), and LMR (*P* = 0.006) were independent prognostic indicators of OS. Moreover, patients of the good LIPI group showed prolonged PFS and OS than those with intermediate/poor LIPI score (*P* < 0.001 and *P* = 0.006, respectively).

**Conclusions:**

Pretreatment dNLR is an independent prognostic indicator of both PFS and OS in stage IV NSCLC patients treated with PD-1 inhibitors. Pretreatment LIPI, combining dNLR > 3 and LDH>ULN, was correlated with worse outcome for stage IV NSCLC patients treated with ICI. High NLR, high dNLR, low LMR, and low ALB at baseline might be useful as an early predictive biomarker of benefit.

## 1. Introduction

Lung cancer is the most frequent malignant cancer and the leading cause of cancer-related death worldwide in recent years [1]. According to the expected number of cancer deaths in 2021 [2], almost one-quarter of all cancer-related deaths are due to lung cancer, among which nearly 82% is directly caused by cigarette smoking. Non-small-cell lung cancer (NSCLC) accounts for 85% of lung cancer cases, and most of the NSCLC patients are diagnosed in advanced stage. For these patients, the poor overall survival (OS) and 5-years survival rate are essential issues.

Over the past decades, cytotoxic systemic chemotherapy remains an important treatment for advanced NSCLC. A majority of patients are still suffering due to drug resistance or side effects of chemotherapy. The primary goal of systemic therapy in metastatic NSCLC is to reduce cancer-related symptoms and to prolong survival time, with a concurrent goal to improve quality of life [3]. The advances of treatment in NSCLC have been greatly improved recently by further understanding of the pathogenic genomic alterations of NSCLC [4], the development of novel drugs, and biomarker-based evidence to identify patients most probably to respond to immune checkpoint blockade therapy.

The immunotherapy revolution, especially the development of immune checkpoint inhibitors (ICIs), has dramatically changed the landscape of the treatment paradigm of advanced NSCLC [5, 6]. The immune checkpoint inhibitors (ICIs) basically include anti-programmed cell death 1 (PD-1) antibodies and anti-programmed cell death 1 ligand 1 (PD-L1) antibodies. These drugs are thought to be functioning by stimulating cell-mediated immunity to recognize and destroy cancer cells and acting or modulating and targeting relevant immune resistance in tumor microenvironment [3, 7, 8]. Although PD-L1 expression is a potential biomarker of the therapeutic response to ICIs, it remains controversial whether it is an optimal predictor. Based on the FDA approvement, nivolumab and pembrolizumab (anti-PD-1 antibody) and atezolizumab (an anti-PD-L1 antibody) are granted as the second-line treatment of advanced NSCLC on the basis of improvements in OS versus docetaxel [9–11]. As a fully human and monoclonal anti-PD1 antibody, nivolumab was the first PD-1 inhibitor which demonstrated meaningful activity in NSCLC. According to the five-year outcome of the phase III trials (CheckMate 017 and CheckMate 057) [12], nivolumab continued to demonstrate the improvement in overall survival (OS) than docetaxel in previously treated advanced NSCLC patients. Besides, five-year OS for advanced NSCLC patients treated with pembrolizumab [13] (KEYNOTE-001) also implicated sufficient antitumor effect and high 5-year OS rates. In the first-line setting, both nivolumab and pembrolizumab have indicated durable antitumor activity and favorable tolerance than platinum-based doublet chemotherapy in patients without EGFR/ALK aberrations and variably PD-L1-enriched patient populations. In KEYNOTE 024 study [14], pembrolizumab significantly improved progression-free survival (PFS) and OS in advanced NSCLC patients with PD-L1 TPS ≥ 50%. On contrast, durvalumab significantly prolonged PFS in unselected patients with stage III NSCLC in the PACIFIC trial [15]. Consequently, immunotherapy biomarkers such as PD-L1 help to enrich clinical benefit but unable to guarantee the benefit or exclude inappropriate patients.

Tumor mutational burden (TMB) is increasingly serving as an alternative predictor of clinical benefit in immunotherapy [16]. TMB is defined as the number of somatic mutations per megabase of an interrogated genomic sequence [17]; it can be assessed by next-generation sequencing (NGS) to quantify the number of nonsynonymous mutations in the entire exome or defined genome [18]. The limitation of expensive cost and time consuming made it difficult to incorporate into clinical practice. Nevertheless, although TMB might be a perfect response biomarker to improve the predictive accuracy for immunotherapy outcomes [16, 17, 19, 20], the prognostic value of TMB still remains uncertain.

The advanced NSCLC consists of IIIB/IIIC stage and IV stage patients, which varies in therapeutic effect, prognosis, and overall survival time. An important unmet need in immunotherapy is to identify predictive factors that may help select patients who are more likely to benefit from ICI, especially the IV stage NSCLC patients. Serum inflammatory biomarkers such as neutrophil-to-lymphocyte ratio (NLR), platelet-to-lymphocyte ratio (PLR), lymphocyte-to-monocyte ratio (LMR), and advanced lung cancer inflammation index (ALI) have been explored as predictive or prognostic factors and the treatment monitoring in NSCLC patients treated with chemotherapy or ICIs [21–25]. However, many indicators such as age, ECOG score, treatment diversification, or drug differences could influence the treatment response or prognosis. Hence, it is important to identify biomarkers helping to provide the most benefit from treatment with minimal risk of toxicity.

In this research, we evaluated the predictive and prognostic significance of biomarkers (NLR, dNLR, LMR, PAB, and LIPI scores) in stage IV NSCLC patients treated with PD-1 inhibitors. We also assessed which biomarker was most specific.

## 2. Materials and Methods

### 2.1. Patients

We conducted a retrospective analysis and enrolled 85 patients who were cytological or histological diagnosed as stage IV NSCLC and treated with anti-PD-1 antibody between June 2018 and Dec 2019 at the Affiliated Tumor Hospital of Nantong University. Inclusion criteria include the following: (1) aged ≥18 years, (2) pathologically confirmed stage IV NSCLC (according to the 8th version of the International Association for the Study of Lung Cancer TNM Staging System), (3) received anti-PD-1 antibody therapy at our hospital from June 2018 to Dec 2019, and (4) complete data collection and follow-up. Patients with a second malignant tumor, severe comorbidities, active systemic inflammatory, autoimmune diseases, and mental disease that could not cooperate with medical treatment were excluded.

The study was performed according to the Declaration of Helsinki and the International Conference on Harmonization Guidelines on Good Clinical Practice. All enrolled patients signed an informed consent form before participating in this study. The Research Ethics Committee of the Affiliated Tumor Hospital of Nantong University approved this retrospective study (2022053).

### 2.2. Treatment and Data Collection

Patients received PD-1 inhibitors or PD-1 combined regimenuntil disease progression, unacceptable drug toxicity, withdrawal, or death. PD-1 inhibitors include pembrolizumab (200 mg every 3 weeks), nivolumab (3 mg/kg every 2 weeks), sintilimab (200 mg every 3 weeks), or toripalimab (240 mg every 3 weeks).

The following data were collected from the medical records: age, sex, smoking history, Eastern Cooperative Oncology Group Performance Status (ECOG score), clinical stage, pathology, biopsy site and method, TNM stage, metastatic sites, EGFR mutation status, PD-L1 expression, line of therapy, treatment regimen and response, and survival outcome (PFS and OS). Blood test results within three weeks prior to the first administration of anti-PD-1 antibody were collected and recorded into the database. The baseline peripheral blood data include total white blood cell concentration (WBC), absolute neutrophil count (ANC), absolute lymphocyte count, total lymphocyte count, platelet count (PLT), monocyte count, hemoglobin concentration (HGB), lactate dehydrogenase (LDH), alanine aminotransferase (ALT), aspartate aminotransferase (AST), serum albumin (ALB) level, serum prealbumin (PAB) level, serum globulin (GLO), carcinoembryonic antigen (CEA), cytokeratin-19 fragments (CYFRA 21-1), neuron-specific enolase (NSE), and squamous cell carcinoma antigen (SCC).

### 2.3. Evaluation of Treatment Response

Each patient was evaluated for treatment efficacy after 6-8 weeks after initial treatment. According to the Response Evaluation Criteria in Solid Tumors (RECIST 1.1 criteria) [26], treatment response was divided into four categories: complete response (CR), partial response (PR), stable disease (SD), and progressive disease (PD). PFS was defined as the time from enrollment to the date of PD or to the end of follow-up (31/08/2021). Overall survival data were obtained from medical records and manual follow-up. Follow-up visits were scheduled for every 3 months during the treatment until death or loss of the visit.

### 2.4. Evaluation of the NLR, LMR, dNLR, and LIPI Score

Based on the baseline peripheral blood data, we calculated NLR, LMR, and dNLR separately. Neutrophil-to-lymphocyte ratio (NLR) was calculated as the ratio of absolute neutrophil count (ANC) divided by absolute lymphocyte count. Lymphocyte-to-monocyte ratio (LMR) was calculated as the ratio of absolute lymphocyte count divided by the absolute monocyte count. The derived NLR (dNLR) was calculated as [ANC/(WBC − ANC)]. The lung immune prognostic index (LIPI) score was defined on the basis of dNLR and LDH level as previously described. LIPI score was divided in three subsets of scores, good, intermediate, and poor LIPI, based on the following cut-off values: dNLR ≤ 3 and LDH ≤ upper limit of normal (ULN), dNLR > 3 or LDH > ULN, and dNLR > 3 and LDH > ULN.

### 2.5. PD-L1 Tumor Expression and Driver Oncogene Mutation Analysis

Patients' relevant data containing PD-L1 tumor expression status and driver gene mutation status (epidermal growth factor receptor (EGFR) and anaplastic lymphoma kinase (ALK)) were extracted from medical records. Immunohistochemical staining (IHC) of PD-L1 expression was performed using pharmDx antibody (clone 22C3, Dako, Carpinteria, CA, USA) following the manufacturer's instructions. EGFR status was determined in tumor samples using the peptide nucleic acid-locked nucleic acid polymerase chain reaction clamp method (AmoyDx, Xiamen, China). ALK status was assessed in tumor tissue using fluorescence in situ hybridization (FISH) with Vysis ALK Break Apart FISH Probe Kit (Abbott Molecular, Des Plaines, IL, USA).

### 2.6. Statistical Analysis

All statistical analyses were performed using SPSS 25.0 (SPSS Inc., Chicago, IL, USA) and GraphPad Prism 8.0 (GraphPad Inc., San Diego, CA, USA). The receiver operating characteristic (ROC) curves were constructed to calculate all indicators included above. The area under the ROC curve (AUC) and asymptotic 95% confidence interval were calculated and recorded. Then, we determined which of the indicators might be predictive markers and defined the cut-off values with maximum sensitivity and specificity. The optimal cut-off values and prognostic roles of biomarkers were identified according to the ROC curves and Youden's index. A chi-square test was performed to compare baseline clinical characteristics. The Cox regression model was conducted to evaluate the predictive factors for PFS and OS via the univariate and multivariate analysis. PFS and OS were determined by the Kaplan-Meier method using the log-rank analysis. All statistical tests were performed two-sided, and a *P* value < 0.05 was considered statistically significant.

## 3. Results

### 3.1. Patient Characteristics

The clinical characteristics of the 85 patients with stage IV NSCLC enrolled in this study are listed in Table 1. The median age was 66 years (range 47-80 years), the majority of patients were male (72.9%), current/former smokers (45.9%), and all patients were clinically diagnosed as stage IV NSCLC. The majority of the patients exhibited the ECOG score of 0-1(88.2%), and the pathology showed the LUSC consisted of 43.5% compared with LUAD (56.5%).

According to the biopsy site, most of the tissues were obtained from the primary lung tumor (62.4%) and lymph nodes (22.4%), while in certain challenging cases, we could also get the tissue from pleural/pericardial effusion, subcutaneous nodules, and bone and surrounding soft tissue. Based on the biopsy method, bronchoscope (34.1%) and CT/Doppler ultrasound (43.6%) were the favorite ways physicians; EBUS-TBNA, pleural/pericardial puncture, or lymph node resection can also help to get tissues.

Most of the patients underwent first-line/second-line therapy with PD-1 antibodies (83.5%) and data of PD-L1 tumor expression were available for 37 patients (43.5%). The PD-L1 expressions in these 37 patients were as follows: 7/39 exhibited PD − L1 < 1% and 11/39 exhibited PD-L1 1-49%, while 19/39 showed PD − L1 ≥ 50%. The treatment regimen included mono drug anti-PD1, anti-PD1+chemotherapy, and anti-PD1+chemotherapy+antiangiogenesis. About 21.2% of patients experienced radiation therapy, and the target lesions of radiotherapy are bone, brain, and lymph nodes. The median values of NLR, dNLR, LDH, LMR, PAB, and ALB were 4.39 (range: 0.89-24.28), 2.9 (range: 0.72-12.75), 219 U/L (range: 23-816), 2.71 (range: 0.44-10.0), 40.5 g/L (range: 20.3-48.9), and 19.3 g/L (range: 2.6-47), respectively.

### 3.2. Tumor Response and Survival Outcome

All 85 patients were evaluated for the tumor response of PD-1-based treatment. The ORR was 42.4% (36/85) and DCR was 68.2% (58/85). The median OS and PFS were 20.0 months (range: 8.0-54 months) and 7.0 months (range: 2.0-25 months), respectively.

### 3.3. Receiver Operating Characteristic (ROC) Curves of Indicators

**Figure 1:**
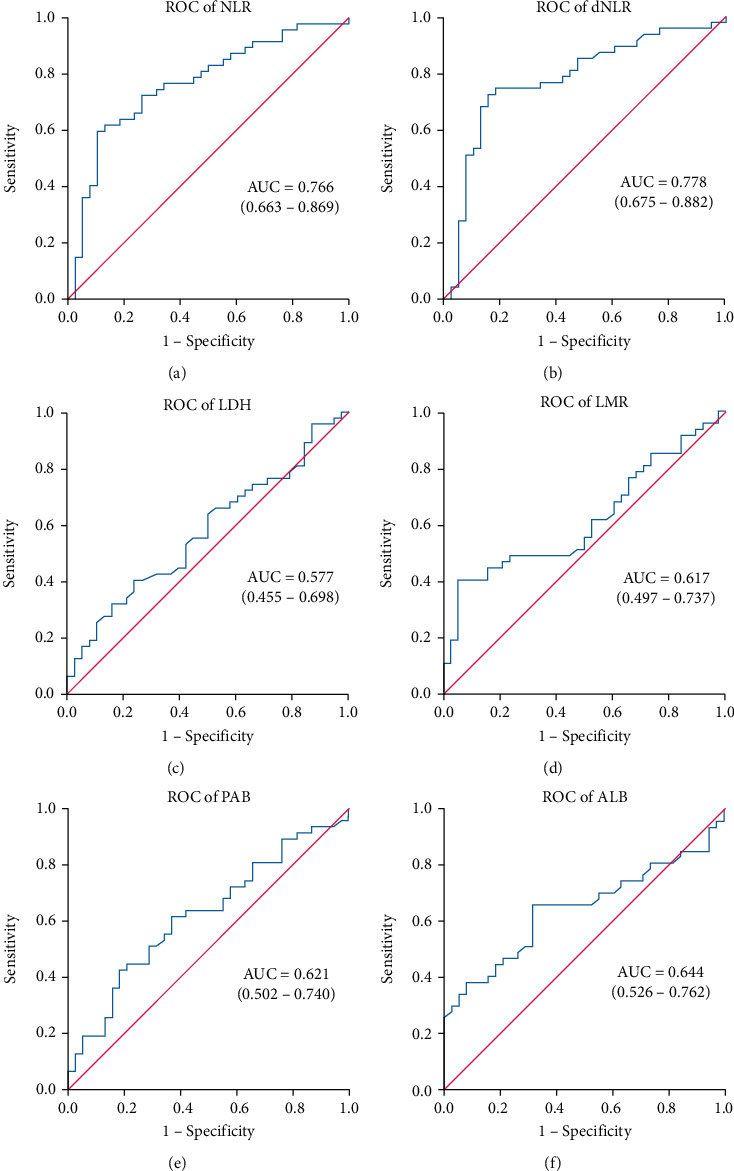
ROC of NLR, dNLR, LDH, LMR, PAB, and ALB.

The receiver operating characteristic (ROC) curves were constructed to access all indicators collected above (Figure S1). The area under the ROC curve (AUC) and asymptotic 95% confidence interval were calculated and recorded separately (Figure S2). Based on AUC and clinical value, we determined to access whether NLR, dNLR, LDH, LMR, PAB, and ALB might be potential predictive markers and calculated the cut-off value of the indicators (Figure 1). Area under the curve (AUC) of NLR, dNLR, LDH, LMR, PAB, and ALB were 0.766, 0.778, 0.577, 0.617, 0.621, and 0.644, respectively. Based on the ROC curves and Youden's index, the cut-off values were 5 for NLR, 3 for dNLR, 210 for LDH, 1.8 for LMR, 35 for ALB, and 17 for PAB, respectively.

### 3.4. The Relationship between Tumor Response and Clinical Characteristics in Stage IV NSCLC Patients Treated with PD-1 Inhibitors

We examined the relationship between tumor response and clinical characteristics in stage IV NSCLC patients. As shown in Table 2, the significant indicators related to ORR were NLR (*P* = 0.02), dNLR (*P* = 0.002), LMR (*P* = 0.03), and ALB (*P* = 0.005), while the indicators related to DCR were NLR (*P* < 0.001), dNLR (*P* < 0.001), LMR (*P* = 0.008), ALB (*P* = 0.045), and LIPI score (*P* = 0.012).

### 3.5. Univariate and Multivariate Analyses of Biomarkers for PFS and OS

The univariate Cox proportional hazard analyses indicated that NLR (≥5 vs. <5, *P* < 0.001), dNLR (≥3 vs. <3, *P* < 0.001), LMR (≥1.8 vs. <1.8, *P* = 0.001), ALB (≥35 vs. <35 g/L, *P* < 0.001), and LIPI (good vs. moderate/poor, *P* = 0.019) were significantly associated with PFS (Table 3). Therefore, they were included in the multivariate analyses which revealed that dNLR (HR = 2.946, 95% CI = 1.615 − 5.373; *P* < 0.001) and ALB level (HR = 0.514, 95% CI = 0.279 − 0.947; *P* = 0.033) were the independent prognostic indicators of PFS in stage IV NSCLC patients treated with PD-1 inhibitors.

Similarly, the univariate Cox proportional hazard analyses also revealed that age (≥70 y vs. <70 y, *P* = 0.02), liver metastasis (yes vs. no, *P* = 0.008), NLR (≥5 vs. <5, *P* = 0.027), dNLR (≥3 vs. <3, *P* < 0.001), LMR (≥1.8 vs. <1.8, *P* = 0.001), ALB (≥35 vs. <35 g/L, *P* = 0.02), PAB (≥17 vs. <17 g/L, *P* = 0.011), and LIPI (good vs. moderate/poor, *P* = 0.038) were significantly associated with OS (Table 3). The multivariate Cox proportional hazard analyses demonstrated that liver metastasis (HR = 4.714, 95% CI = 1.562 − 14.225; *P* = 0.01), NLR (HR = 4.092, 95% CI = 1.407 − 11.899; *P* = 0.01), dNLR (HR = 5.907, 95% CI = 2.101 − 16.610; *P* = 0.001), and LMR (HR = 0.315, 95% CI = 0.138 − 0.721; *P* = 0.006) were the independent prognostic indicators of OS in stage IV NSCLC treated with PD-1 inhibitors.

### 3.6. Prognostic Significance of Serum Biomarkers in Stage IV NSCLC Patients

**Figure 2:**
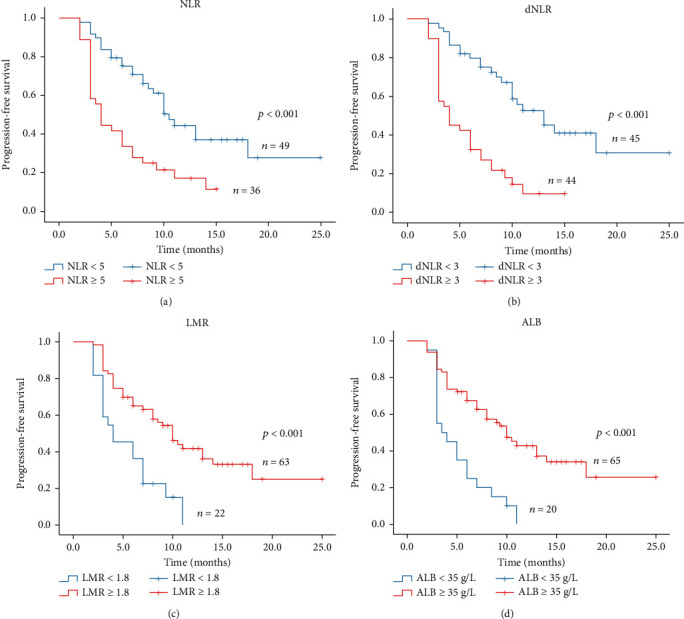
Kaplan-Meier curves of PFS in stage IV non-small-cell lung cancer treated with PD-1 inhibitors.

**Figure 3:**
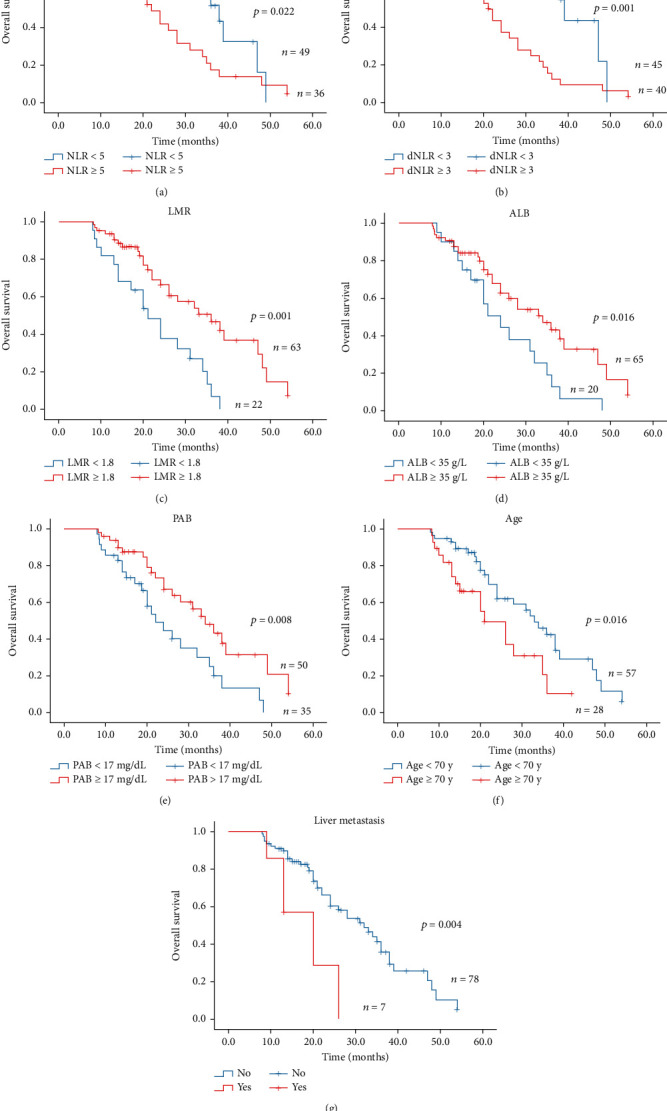
Kaplan-Meier curves of OS in stage IV non-small-cell lung cancer treated with PD-1 inhibitors.

Based on the results of the univariate and multivariate analysis of PFS/OS, we further calculated the Kaplan-Meier curves to evaluate the association between the important indicators and PFS/OS. As indicated in Figures 2 and 3, compared with low NLR group (NLR < 5), high NLR group (NLR ≥ 5) had significantly shorter median PFS (4.0 vs. 10.5 months, *P* < 0.001; Figure 2(a)) and shorter median OS (22.0 vs. 38.0 months; *P* = 0.022; Figure 3(a)). Besides, the high dNLR group (dNLR ≥ 3) had significantly shorter median PFS (4.0 vs. 13.0 months; *P* < 0.001; Figure 2(b)) and OS (21.0 vs. 39.0 months; *P* < 0.001; Figure 3(b)) than the low dNLR (NLR < 5) group. Moreover, high LMR (LMR ≥ 1.8) group suggested shorter median PFS (4.0 vs. 10.0 months; *P* < 0.001; Figure 2(c)) and median OS (21.0 vs. 36.0 months; *P* = 0.001; Figure 3(c)) than low LMR (LMR < 1.8). Meanwhile, the Kaplan-Meier analysis and log-rank test also demonstrated that ALB (Figure 2(d)), PAB (Figure 3(e)), age (Figure 3(f)), and liver metastasis (Figure 3(g)) also helped to predict the survival outcome.

Taken together, the prognostic biomarkers such as NLR (*P* < 0.001), dNLR (*P* < 0.001), LMR (*P* < 0.001), and ALB (*P* < 0.001) might be significant indicators for PFS, while the age (*P* = 0.016), liver metastasis (*P* = 0.005), NLR (*P* = 0.01), dNLR (*P* = 0.001), and LMR (*P* = 0.006) might be promising biomarkers for the prediction of OS.

### 3.7. PFS and OS in Stage IV NSCLC Patients with PD-1 Inhibitor Monotherapy

**Figure 4:**
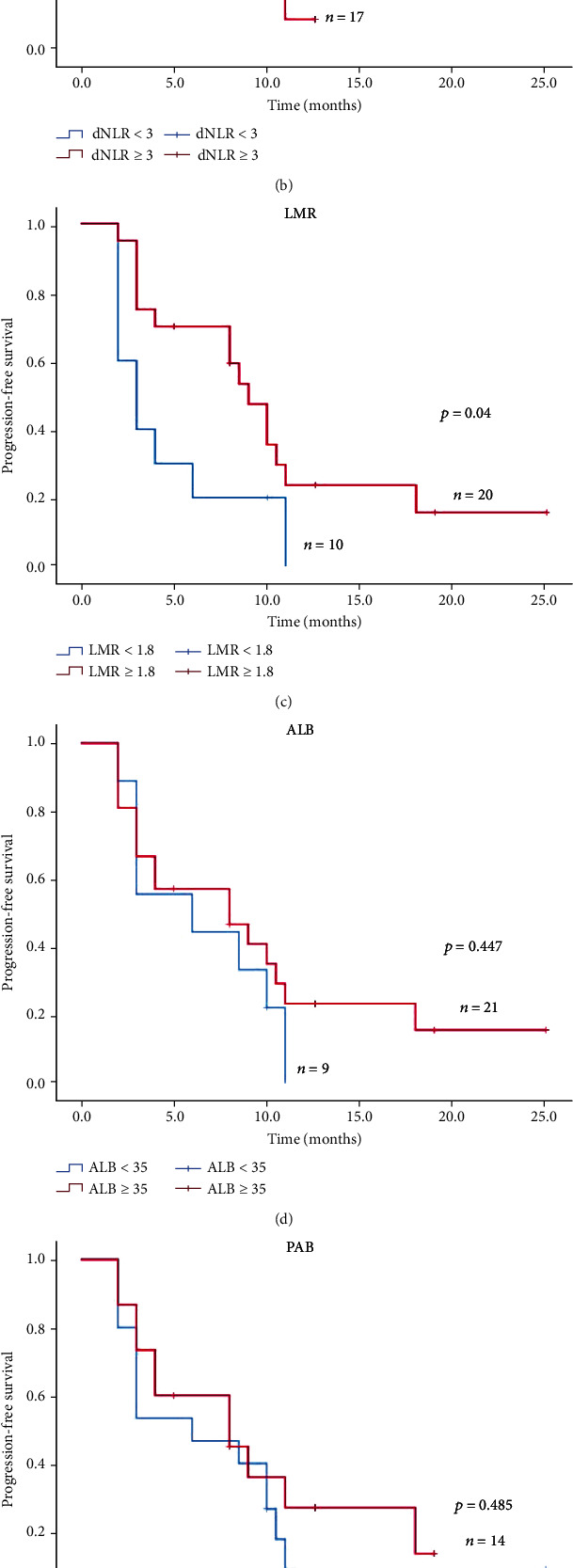
Kaplan-Meier curves of PFS in stage IV NSCLC treated with PD-1 inhibitor monotherapy.

**Figure 5:**
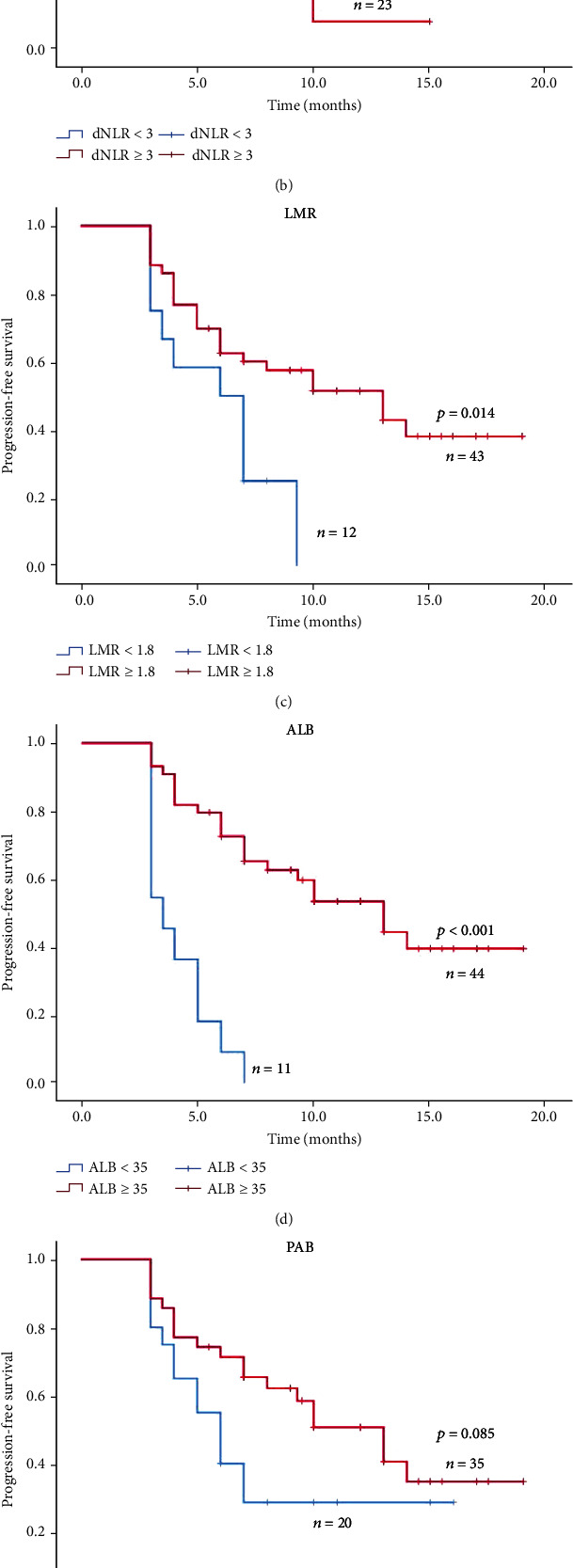
Kaplan-Meier curves of OS in stage IV NSCLC treated with PD-1 inhibitor monotherapy.

In our enrolled patients,30 patients underwent PD-1 inhibitor monotherapy. Then, we analyzed the association of serum biomarkers in patients with PD-1 inhibitor monotherapy individually. As indicated in Figure 4, high NLR group (NLR ≥ 5), high dNLR group (dNLR ≥ 3), and low LMR (LMR < 1.8) group suggested shorter median PFS in PD-1 monotherapy patients (*P* < 0.05), while in Figure 5, only high LMR (LMR ≥ 1.8) group suggested longer median PFS in PD-1 monotherapy patients (*P* < 0.05).

### 3.8. PFS and OS in Stage IV NSCLC Patients with PD-1 Inhibitor Combination Therapy

**Figure 6:**
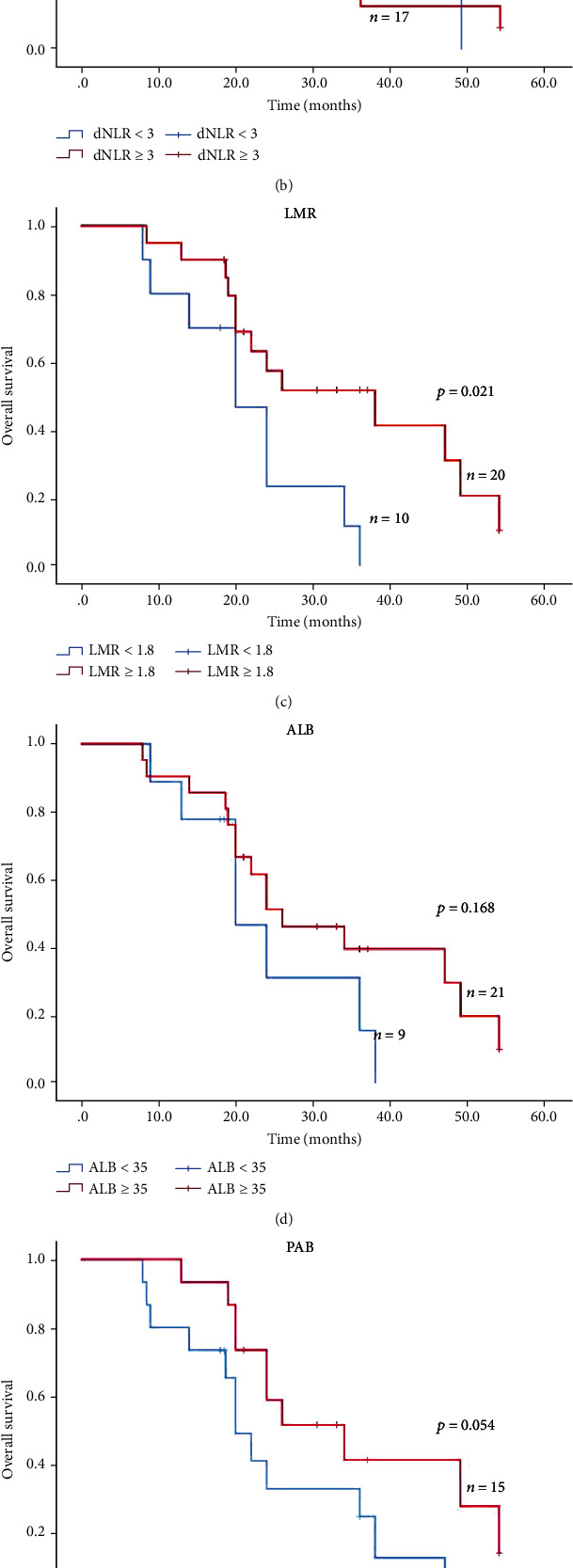
Kaplan-Meier curves of PFS in stage IV NSCLC treated with PD-1 inhibitor combination therapy.

**Figure 7:**
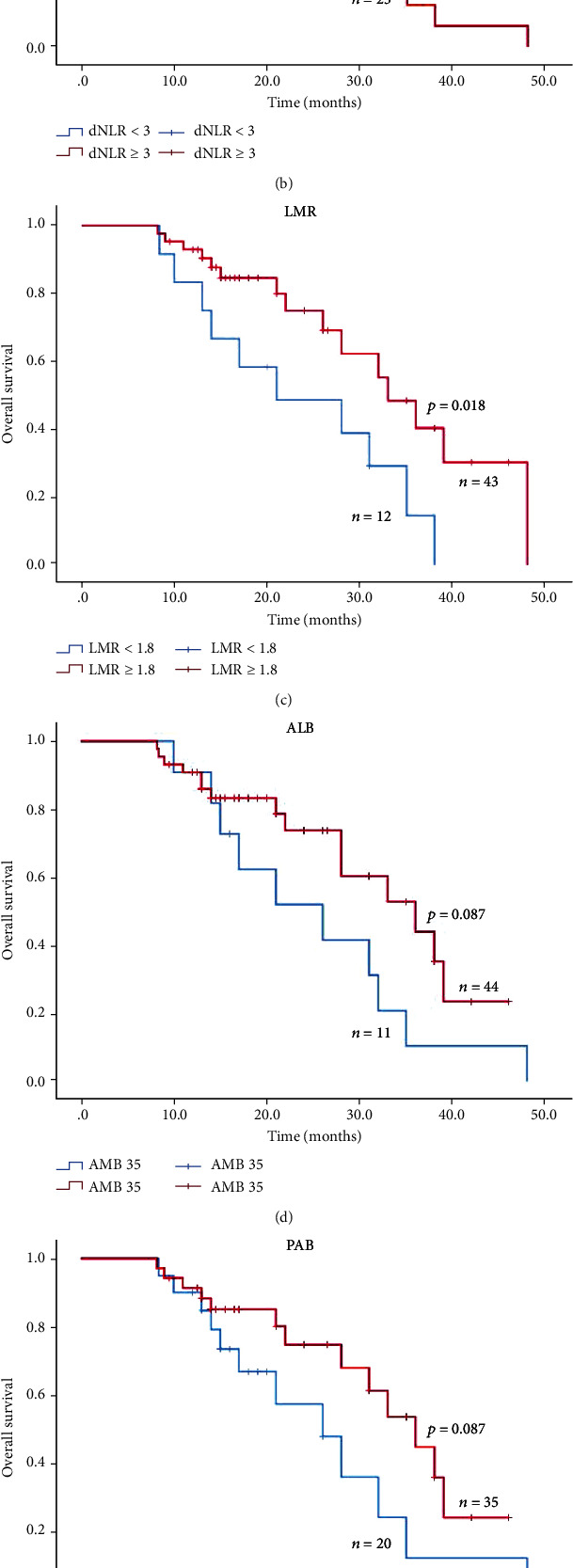
Kaplan-Meier curves of OS in stage IV NSCLC treated with PD-1 inhibitor combination therapy.

We analyzed the association of serum biomarkers in patients with PD-1 inhibitor combination therapy. As indicated in Figure 6, high NLR group (NLR ≥ 5), high dNLR group (dNLR ≥ 3), low LMR (LMR < 1.8), and low ALB (ALB < 35) group suggested shorter median PFS in PD-1 combination therapy patients (*P* < 0.05), while in Figure 7, only high LMR (LMR ≥ 1.8) group suggested longer median PFS in PD-1 monotherapy patients (*P* < 0.05).

### 3.9. Multifactor Model of LIPI Score for Survival Outcome of Stage IV NSCLC Patients Treated with PD-1 Inhibitors

**Figure 8:**
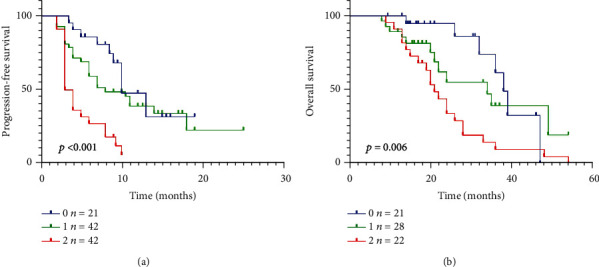
Multifactor model of LIPI score for survival outcome of stage IV NSCLC patients treated with PD-1 inhibitors.

Based on the following cut-off values, dNLR ≤ 3 and LDH ≤ upper limit of normal (ULN), dNLR > 3 or LDH > ULN, and dNLR > 3 and LDH > ULN, LIPI score was divided into three subsets of scores: good (0), intermediate (1), and poor (2) LIPI. Then, we explored the OS and PFS by the multifactor model of LIPI score. As indicated in Figure 8(a), PFS of patients with good LIPI score were significantly longer than those with intermediate/poor LIPI score (*P* < 0.001). Similarly, good LIPI score cohort achieved better overall survival time than the intermediate/poor LIPI score cohort (*P* < 0.006, Figure 8(b)).

## 4. Discussions

This study investigated the predictive and prognostic value of serum biomarkers in stage IV NSCLC patients treated with PD-1 inhibitors. The results revealed that low dNLR or good LIPI score predicts better survival outcomes for these patients, no matter PFS or OS. Further, high dNLR and low ALB level were independent prognostic factors of shorter PFS. Meanwhile, liver metastasis, high NLR, high dNLR, and LMR were independent prognostic indicators of shorter OS. Moreover, prognostic biomarkers such as NLR, dNLR, LMR, and ALB might be significant indicators for PFS, while age, liver metastasis, NLR, dNLR, and LMR might be promising biomarkers for the prediction of OS. Furthermore, in the good LIPI group, PFS and OS were significantly longer. In clinical settings, it is important to use accurate and effective markers to guide clinical treatment and predict prognosis. On the one hand, all these involved serum indicators are easy to obtain and record, so monitoring the indicators above provides a simple and convenient method for clinicians. On the other hand, for certain stage IV non-small-cell lung cancer (NSCLC) patients treated with PD-1 inhibitors, the early prediction of prognosis is definitely of great significance.

A major advancement in cancer treatment is the development of immune checkpoint inhibitors (ICI), which have produced long-lasting responses and improved survival rates in a variety of solid malignancies [17]. However, since most patients receiving ICI treatment did not achieve the expected results, it is necessary to identify predictive biomarkers of ICI response to achieve more clinical benefit, as well as to clarify and overcome the mechanism of treatment resistance. Tumor mutational burden is an indicator controversial. Previously, a study reported that tTMB correlates with bTMB and bTMB helps to identify patients who derive clinically significant improvements in PFS from atezolizumab in second-line or posterior treatment in NSCLC [19]. In terms of determining tumor heterogeneity, the outline of simultaneous genomic changes in NSCLC may be more influential than obvious mutations in oncogenic drivers. Based on current clinical research evidence, cooccurring genomic alterations could further affect the clinical response to ICIs. A recent research implicated that the diversity of KRAS-mutant lung adenocarcinomas (LUADs) is associated with different characters, such as KRAS dependency, immunogenicity, and STK11/KEAP1 comutations [6, 27]. Such features may serve as biomarkers for drug sensitivity prediction, especially in immunotherapy. Another study [28] investigated the prognostic value of STK11/KEAP1 mutations in an observational real-world lung adenocarcinoma cohort, and the results suggested that STK11/KEAP1 mutations are prognostic, not predictive biomarkers for immunotherapy. Besides, current studies have shown that TP53 and KRAS mutations in LUAD may be a pair of potential predictive factors in guiding anti-PD-1/PD-L1 immunotherapy [29]. All in all, these markers require sufficient tissue or blood samples for testing. Besides, a certain amount of financial support is needed to complete the inspection.

Previous studies have investigated the potential utility of routine blood parameters in the treatment of various tumors. However, there is no uniform boundary value for the emerging biomarkers. In a study evaluating the prognostic value of NLR, PLR, and NLR–PLR score in stage IV GC patients [30], NLR–PLR score showed the value of independently predicted survival outcomes. As for the treatment response for early phase SCLC patients treated with immunotherapy [31], NLR at 6 weeks after initial treatment appears to be a biomarker; the cut-off value of NLR is set as 5. In another research exploring the value of lymphocyte-to-monocyte ratio (LMR) in advanced NSCLC patients who received nivolumab monotherapy [25], the result indicated the rapid increase of LMR (increasement ≥ 10%) was significantly associated with treatment response. Tong et al. conducted a retrospective analysis involving 332 newly diagnosed stage III NSCLC patients [32], which demonstrated that SII (cut-off value 660) is an independent prognostic indicator of poor outcomes for patients. Lung immune prognostic index (LIPI) is also an emerging biomarker that deserves attention. In JAMA Oncology [23, 33], researches demonstrated that baseline LDH levels and dNLR are important prognostic biomarkers for metastatic NSCLC patients. An Italian study [34] reported that the systemic inflammatory biomarkers such as ALI, LDH, NLR, and LIPI score may help the understanding of survival differences in the clinical management of lung neuroendocrine carcinomas. In one study, enrolling patients with advanced hepatocellular carcinoma underwent immunotherapy [35], pretreatment LIPI (dNLR ≥ 3 and/or LDH ≥ ULN) were associated with poor outcomes.

Although the application of serum biomarkers is emerging into clinical practice, there is still controversial on this issue. First of all, some clinicians argued that different treatments or different populations should have their different cut-off values. The difference of the analysis method and the patients included in the study may influence the cut-off value of each study. Secondly, complications including anemia, pneumonia, abnormal hypothyroidism, liver disease, and heart dysfunction may also affect the serum concentrations of the indicators. Given the inevitable interference of these factors in real-world clinical practice, how to avoid interference scientifically and reasonably raises new questions for clinicians. Thirdly, the stage or the metastatic site of the disease may also have a critical effects on the prognosis. For these reasons, we designed this research aimed to evaluate the significance of blood parameters in this specific population of stage IV non-small-cell lung cancer. Our retrospective study revealed that pretreatment dNLR is an independent prognostic indicator of both PFS and OS in stage IV NSCLC patients treated with PD-1 inhibitors. Besides, pretreatment LIPI combining dNLR ≥ 3 and LDH≥ULN was correlated with worse outcome for stage IV NSCLC patients treated with ICI. High NLR, high dNLR, low LMR, and low ALB at baseline might be useful as an early predictive biomarker of benefit.

However, there are still some shortcomings within our investigation. The first one is, as a retrospective analysis of a single center, the sample size is relatively small. In view of the drug approval of checkpoint inhibitors that were first approved by the National Medical Products Administration (NMPA) in China since June 2018, we only included 85 patients with stage IV NSCLC treated with PD-1 inhibitors in detail. In the next step, our research group intends to further expand the sample size and conduct studies in conjunction with multiple clinical centers. In addition, hematology parameters may be affected by some concurrent medications. As far as our study excluded patients with a second malignant tumor, severe comorbidities, active systemic inflammatory, autoimmune diseases, and mental disease, the effect of combination medication is relatively reduced. Finally, this study has not fully elucidated the basic biological mechanisms. Nonetheless, our study provides a simple, economical, and noninvasive method to help clinicians predict the response and prognosis of anti-PD-1 antibodies. Future research will focus on exploring the significance of serum markers in early-stage NSCLC, and the importance of immunotherapy in a distinct populations.

## 5. Conclusions

Pretreatment dNLR is an independent prognostic indicator of both PFS and OS in stage IV NSCLC patients treated with PD-1 inhibitors. Pretreatment LIPI, combining dNLR > 3 and LDH>ULN, is correlated with worse outcome for stage IV NSCLC patients treated with ICI. High NLR, high dNLR, low LMR, and low ALB at baseline might be useful as an early predictive biomarker of benefit.

## Figures and Tables

**Table 1:** Clinicopathological characteristics of stage IV NSCLC patients.

Characteristics (*n* = 85)		*N* (%)
Age		
Median		66
Range		47-80
<70 y		57 (67.1)
≥70 y		28 (32.9)
Sex		
Male		62 (72.9)
Female		23 (27.1)
Smoking		
Current/former		39 (45.9)
No		46 (54.1)
EOCG		
0-1		66 (88.2)
2		19 (11.8)
Stage		
IVA		38 (44.7)
IVB		47 (55.3)
Pathology		
LUSC		37 (43.5)
LUAD		48 (56.5)
Biopsy site		
Primary lung tumor		53 (62.4)
Lymph nodes		19 (22.4)
Liver		2 (2.4)
Pleural/pericardial effusion		6 (7.1)
Subcutaneous nodules		2 (2.4)
Bone and surrounding soft tissue		3 (3.5)
Biopsy method		
Bronchoscope		29 (34.1)
EBUS-TBNA		5 (5.9)
Computed tomography/Doppler ultrasound		37 (43.6)
Pleural/pericardial puncture		7 (8.2)
Lymph node resection		7 (8.2)
T		
T1-2		39 (25.8)
T3-4		46 (54.2)
N		
N1		7 (8.2)
N2		15 (17.6)
N3		63 (74.1)
M		
M1a		30 (35.3)
M1b		9 (10.6)
M1c		46 (54.1)
Metastatic site		
Liver		
Yes		7 (8.2)
No		78 (91.8)
Bone		
Yes		42 (49.4)
No		43 (50.6)
Brain		
Yes		7 (8.2)
No		78 (91.8)
Pleural/lung		
Yes		44 (51.8)
No		41 (48.2)
Other sites		
Yes		50 (58.8)
No		35 (41.2)
EGFR		
Wild		75 (88.2)
Mutant		10 (11.8)
PD-L1(22C3)		
<1%		7 (8.2)
1-49%		11 (12.9)
≥50%		19 (22.4)
Unknown		48 (56.5)
Line of treatment		
1st		32 (37.6)
2nd		39 (45.9)
≥3		14 (16.5)
Treatment regimen		
Mono drug anti-PD1		30 (35.3)
Anti-PD1+chemotherapy		49 (57.6)
Anti-PD1+chemotherapy+antiangiogenesis		6 (7.1)
Radiation therapy	Yes	18 (21.2)
	No	67 (78.8)
NLR	Median (mean)	4.39 (5.78)
	Range	0.89-24.28
dNLR	Median (mean)	2.9 (3.3)
	Range	0.72-12.75
LDH	Median (mean)	219 (233.68)
	Range	23-816
LMR	Median (mean)	2.71 (3.07)
	Range	0.44-10.0
ALB	Median (mean)	40.5 (38.95)
	Range	20.3-48.9
PAB	Median (mean)	19.3 (20.17)
	Range	2.6-47

**Table 2:** Association of ORR/DCR and clinical characteristics.

Characteristics (*n* = 85)		*N* (%)	Overall response rate (ORR)		Disease control rate (DCR)	
			% (*N*)	*P* value	% (*N*)	*P* value
Overall			42.4% (36/85)		68.2% (58/85)	
Gender	Male	62 (72.9)	40.3% (25/62)	0.534	66.1% (41/62)	0.493
	Female	23 (27.1)	47.8% (11/23)		73.9% (17/23)	
Age	<70 y	57 (67.1)	42.1% (24/57)	0.947	70.2% (40/57)	0.584
	≥70 y	28 (32.9)	42.9% (12/28)		64.3% (18/28)	
Smoking	No	46 (54.1)	47.8% (22/46)	0.267	69.6% (32/46)	0.775
	Yes	39 (45.9)	35.9% (14/39)		66.7% (26/39)	
Histology	LUSC	37 (43.5)	40.5% (15/37)	0.767	62.2% (23/37)	0.291
	LUAD	48 (56.5)	43.8% (21/48)		72.9% (35/48)	
Stage	IVA	38 (44.7)	42.1% (16/38)	0.967	65.8% (25/38)	0.663
	IVB	47 (55.3)	42.6% (20/47)		70.2% (33/47)	
Line of treatment	<3	71 (83.5)	46.5% (33/71)	0.083	71.8% (51/71)	0.109
	≥3	14 (16.5)	21.4% (3/14)		50.0% (7/14)	
NLR	<5	49 (57.6)	53.1% (26/49)	0.02	85.7% (42/49)	<0.001
	≥5	36 (42.4)	27.8% (10/36)		44.4% (16/36)	
dNLR	<3	45 (52.9)	57.8% (26/45)	0.002	88.9% (40/45)	<0.001
	≥3	40 (47.1)	25.0% (10/40)		45.0% (18/40)	
LDH	<210	39 (45.9)	41.0% (16/39)	0.82	74.4% (29/39)	0.264
	≥210	46 (54.1)	43.5% (20/46)		63.0% (29/36)	
LMR	<1.8	22 (25.9)	22.7% (5/22)	0.03	45.5% (10/22)	0.008
	≥1.8	63 (74.1)	49.2% (31/63)		76.2% (48/63)	
ALB	<35	20 (23.6)	15.0% (3/20)	0.005	50.0% (10/20)	0.045
	≥35	65 (76.5)	50.8% (33/65)		73.8% (48/65)	
PAB	<17	35 (41.1)	34.3% (12/35)	0.208	62.9% (22/35)	0.373
	≥17	50 (58.8)	48.0% (24/50)		72.0% (36/50)	
LIPI	Intermediate/poor	21 (24.7)	52.4% (11/21)	0.284	90.5% (19/21)	0.012
	Good	64 (75.3)	39.1% (25/64)		60.9% (39/64)	

**Table 3:** Univariate and multivariate analyses of biomarkers for PFS and OS.

		PFS				OS			
		Univariate analysis		Multivariate analysis		Univariate analysis		Multivariate analysis	
		HR (95% CI)	*P* value	HR (95% CI)	*P* value	HR (95% CI)	*P* value	HR (95% CI)	*P* value
Gender	Female vs. male	0.679	0.235			0.418	0.021		
		0.359-1.286				(0.199-0.877)			
Age	≥70 y vs. <70 y	1.193	0.526			2.093	0.02		
		0.692-2.057				(1.125-3.895)			
ECOG	≥2 vs. <2	1.323	0.391			1.791	0.186		
		0.698-2.509				(0.756-4.242)			
Smoking	Yes vs. no	1.294	0.332			1.667	0.089		
		0.768-2.180				(0.926-3.002)			
Histology	LUAD vs. LUSC	0.981	0.942			0.577	0.066		
		0.580-1.657				(0.321-1.037)			
Stage	IVA vs. IVB	0.786	0.364			0.821	0.507		
		0.467-1.323				(0.458-1.471)			
EGFR	Mutant vs. wild	1.253	0.557			0.686	0.339		
		0.590-2.659				(0.316-1.487)			
Liver metastasis	Yes vs. no	1.978	0.12			3.696	0.008	4.714	0.005
		0.838-4.667				(1.396-0.786)		1.562-14.225	
Bone metastasis	Yes vs. no	0.713	0.205			0.666	0.175		
		0.422-1.203				(0.371-1.198)			
Brain metastasis	Yes vs. no	1.063	0.897			0.908	0.854		
		0.423-2.673				(0.324-2.544)			
Pleural/lung metastasis	Yes vs. no	1.052	0.849			0.967	0.912		
		0.625-1.770				(0.538-1.738)			
Other sites	Yes vs. no	0.894	0.678			1.057	0.854		
		0.528-1.515				(0.588-1.899)			
Line of treatment	≥3 vs. <3	1.646	0.159			0.878	0.719		
		0.823-3.292				(0.432-1.784)			
Radiation therapy	Yes vs. no	1.019	0.955			1.011	0.975		
		0.535-1.941				(0.511-1.999)			
NLR	≥5 vs. <5	0.385	<0.001			1.963	0.027	4.092	0.01
		0.226-0.656				1.081-3.563		1.407-11.899	
dNLR	≥3 vs. <3	3.58	<0.001	2.946	<0.001	3.186	<0.001	5.907	0.001
		2.039-6.284		1.615-5.373		1.669-6.082		2.101-16.610	
LDH	≥210 vs. <210	1.146	0.611			1.248	0.456		
		0.678-1.936				0.697-2.234			
LMR	≥1.8 vs. <1.8	0.367	0.001			0.367	0.001	0.315	0.006
		0.206-0.654				0.198-0.678		0.138-0.721	
ALB	≥35 vs. <35	0.335	<0.001	0.514	0.033	0.488	0.02		
		0.118-0.596		0.279-0.947		0.267-0.893			
PAB	≥17 vs. <17	0.592	0.05			0.467	0.011		
		0.350-1.000				0.259-0.841			
LIPI	Good vs. intermediate/poor	2.276	0.019			2.365	0.038		
		1.146-4.521				1.051-5.325			

## Data Availability

Data is owned and saved by the Affiliated Tumor Hospital of Nantong University and are available on request to the corresponding author. For researchers meeting the criteria for access to confidential data, please contact the following E-mail address: xiaodongzhang@csco.ac.cn.

## Supplementary Materials

Supplementary 1 Figure S1: ROC of all indicators.

Supplementary 2 Figure S2: AUC and 95% CI of indicators.
