# Supplementary figures and images for: Clinical Significance of Serum Biomarkers in Stage IV Non-Small-Cell Lung Cancer Treated with PD-1 Inhibitors: LIPI Score, NLR, dNLR, LMR, and PAB
Source: Dis Markers. 2022 Jul 30;2022:7137357. doi: 10.1155/2022/7137357 (PMC9357262; doi:10.1155/2022/7137357)

**ROC of Indicators**

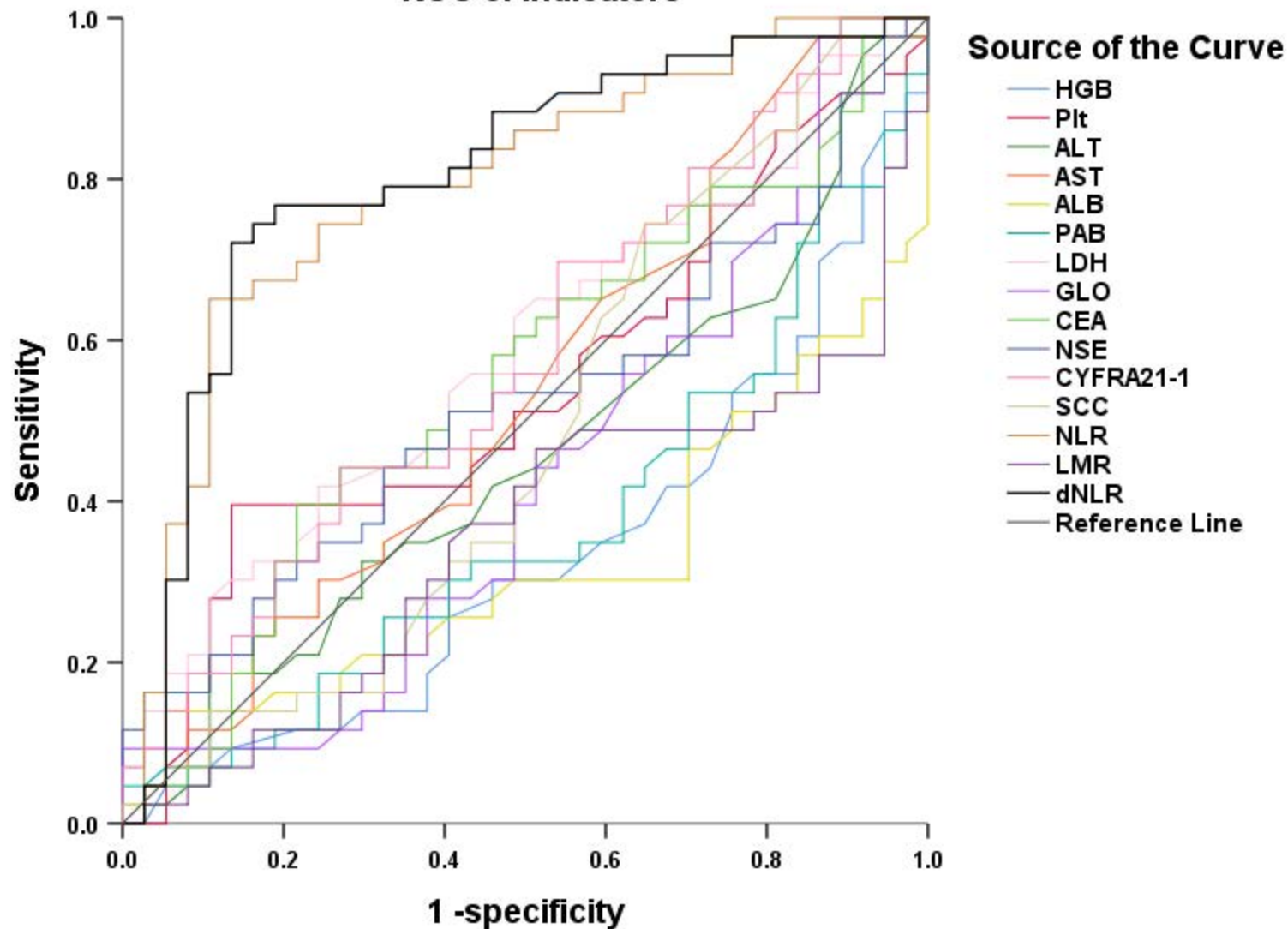

Supplement: Supplementary 1 — Figure S1: ROC of all indicators. [file 7137357.f1.pdf]

A

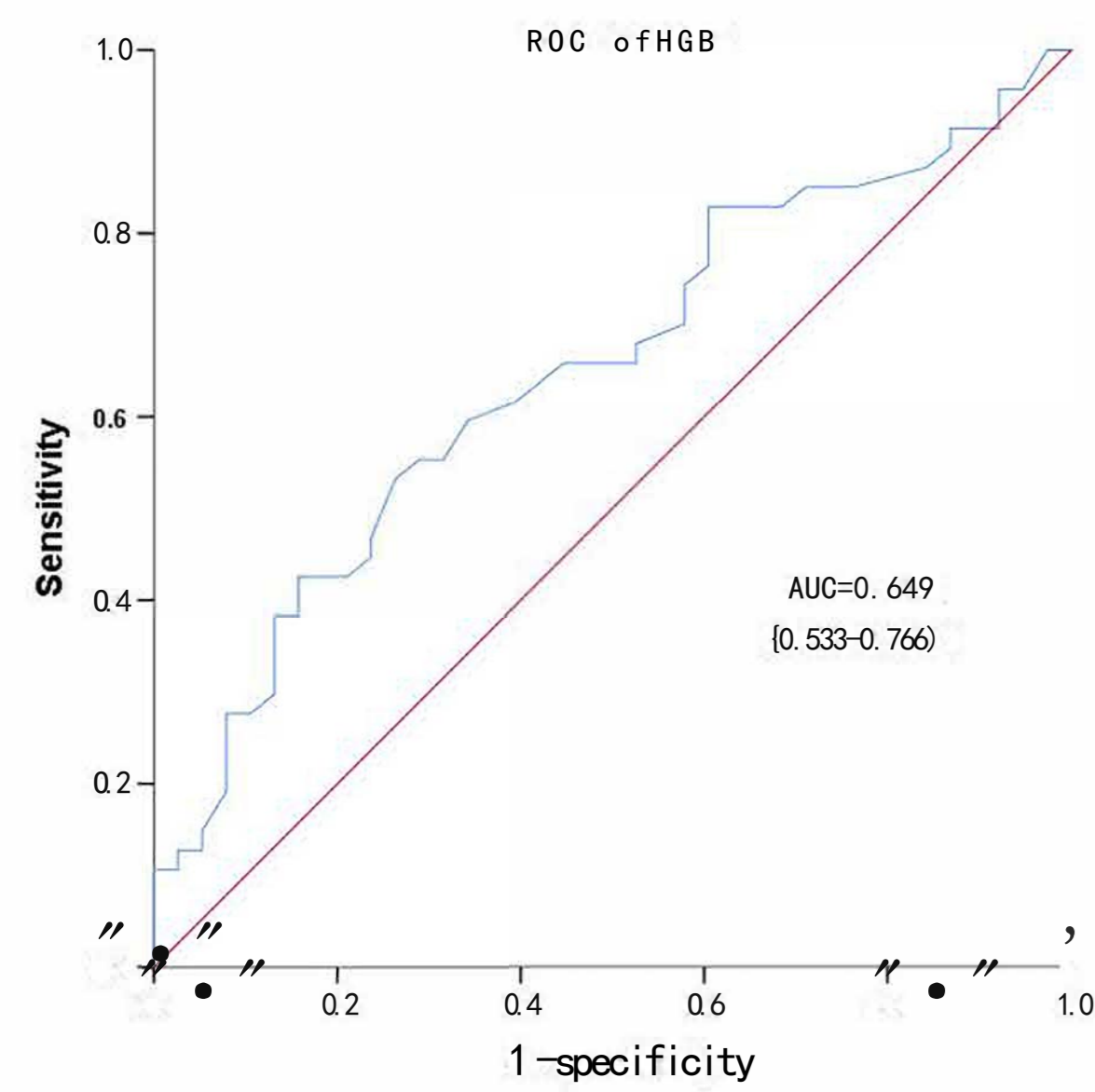

B

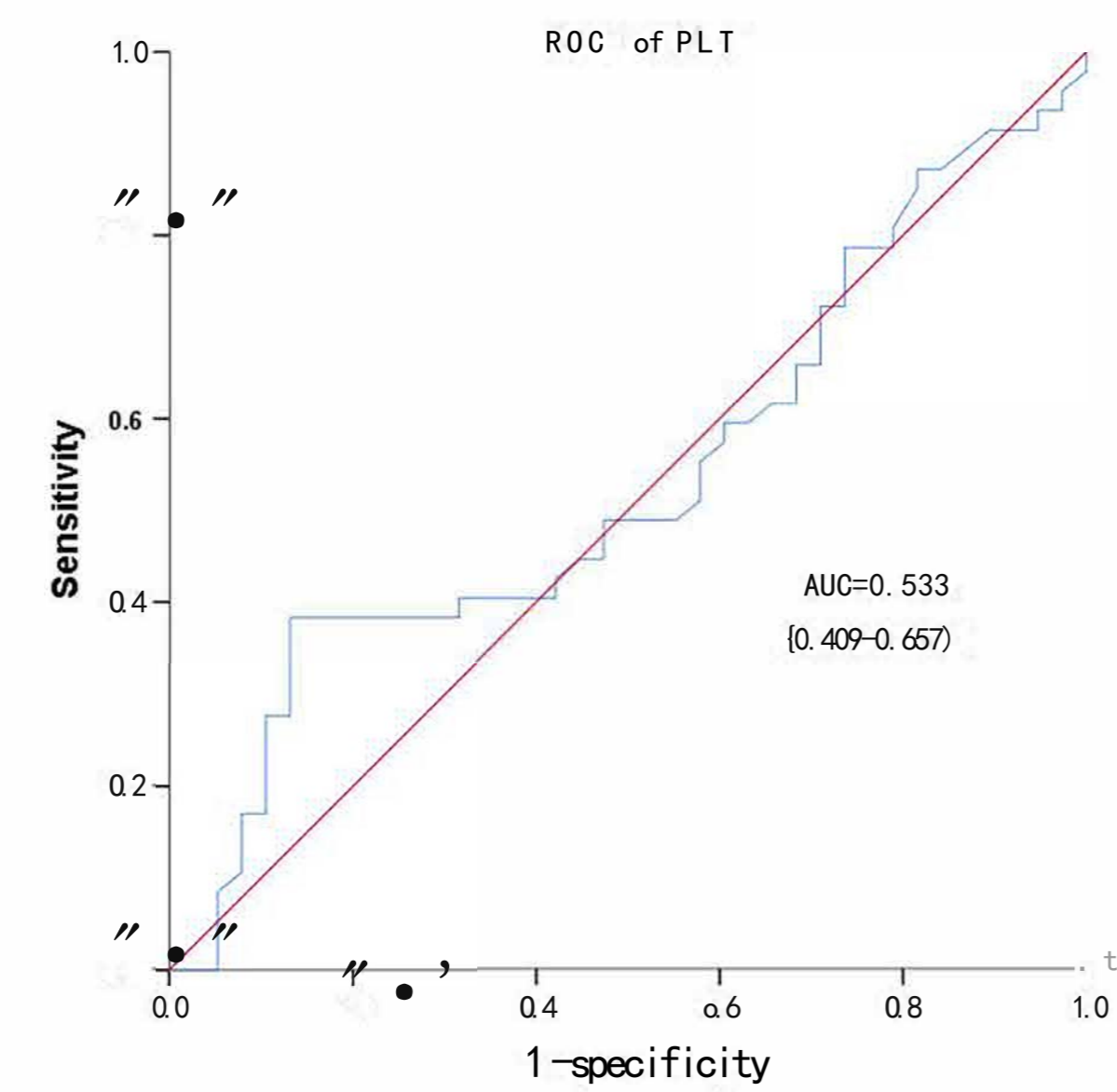

C

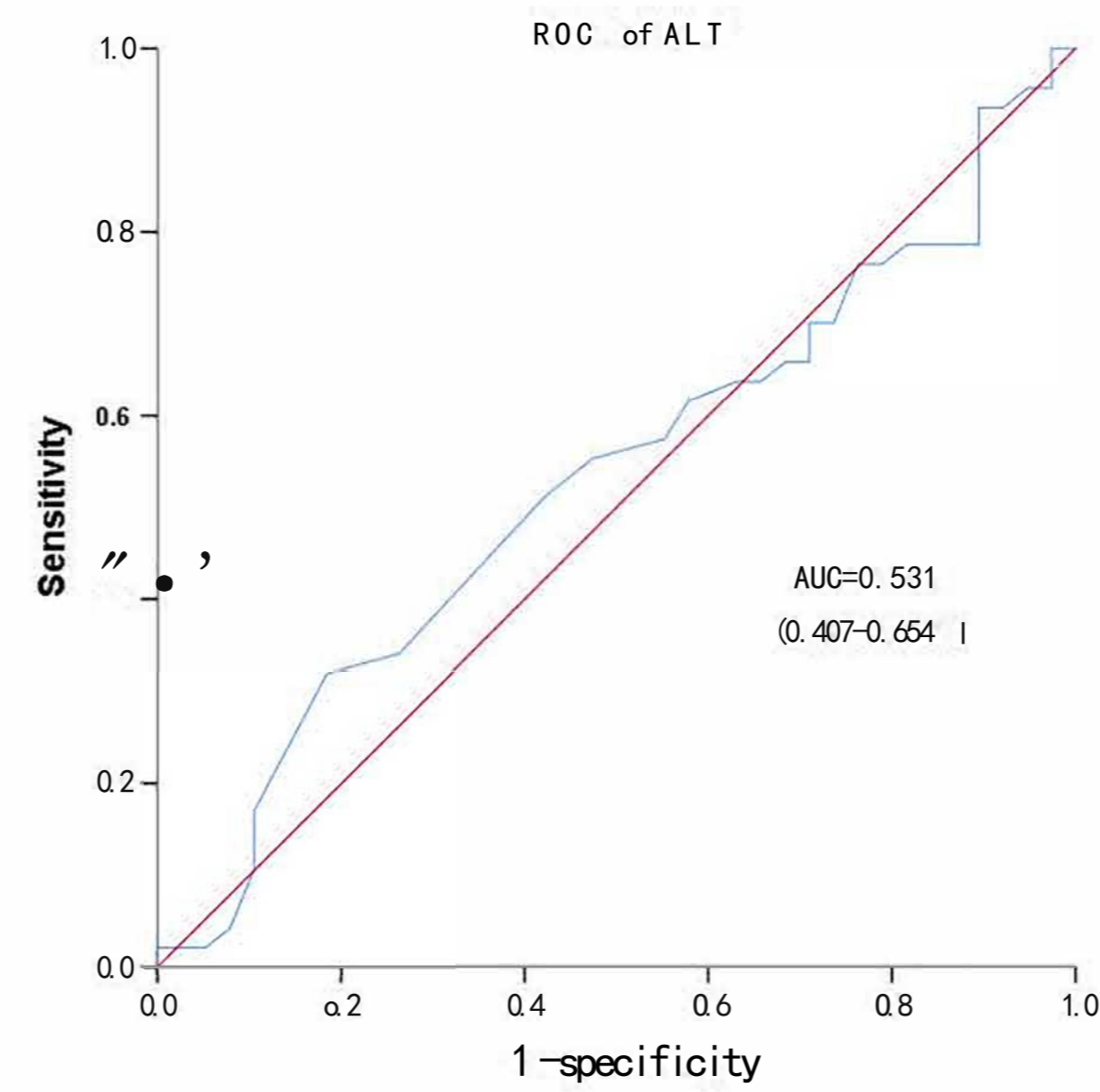

D

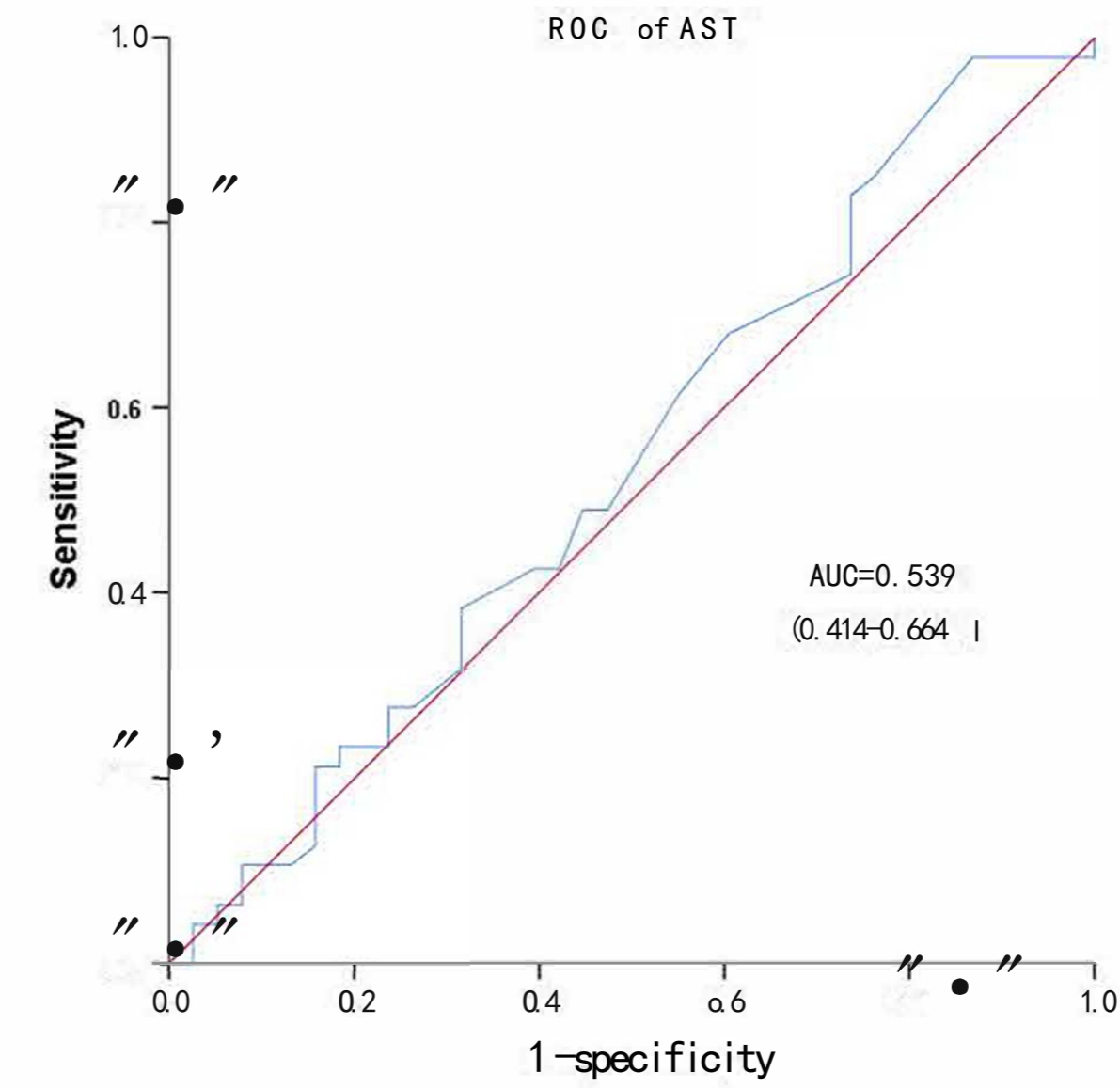

E

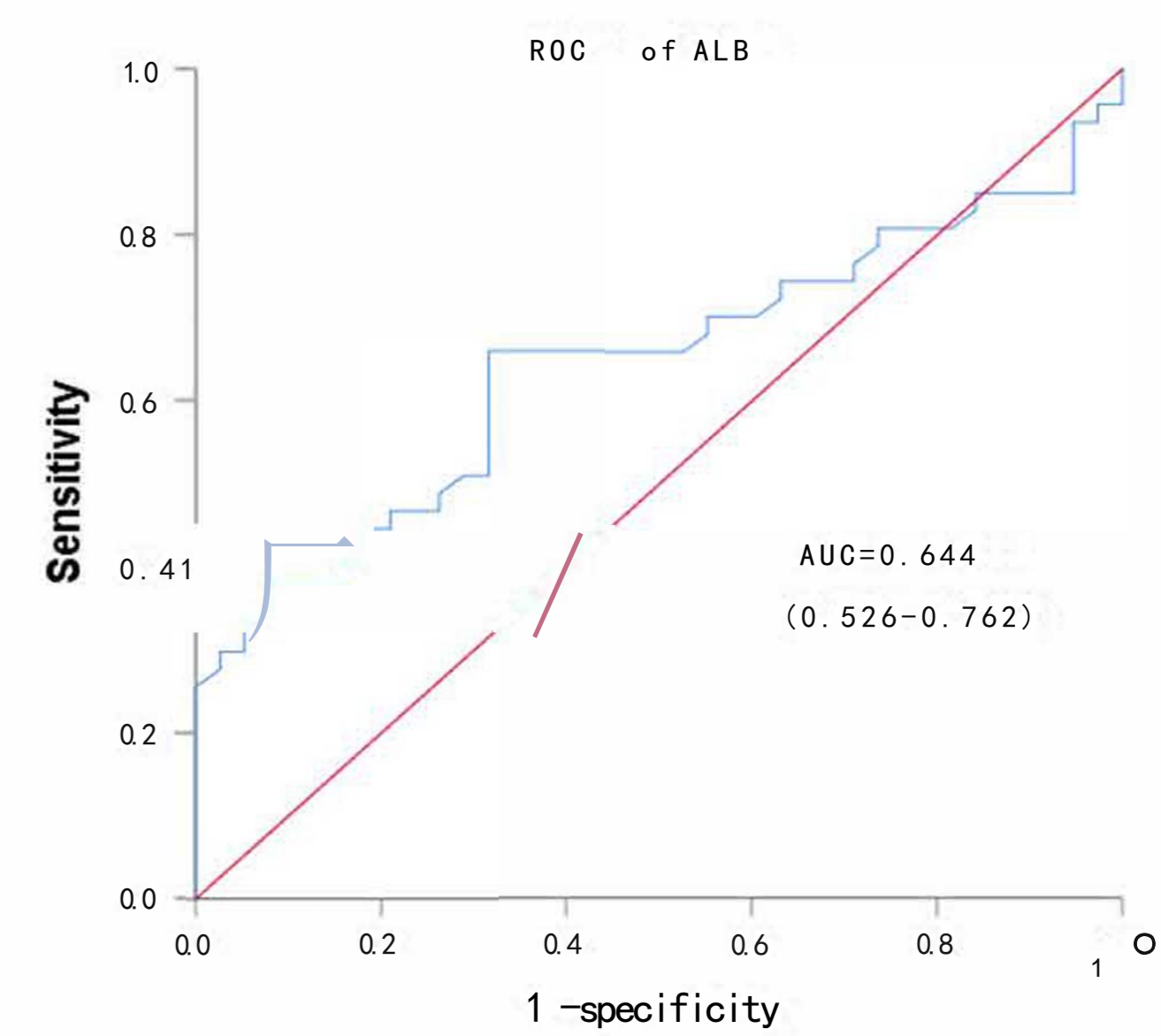

F

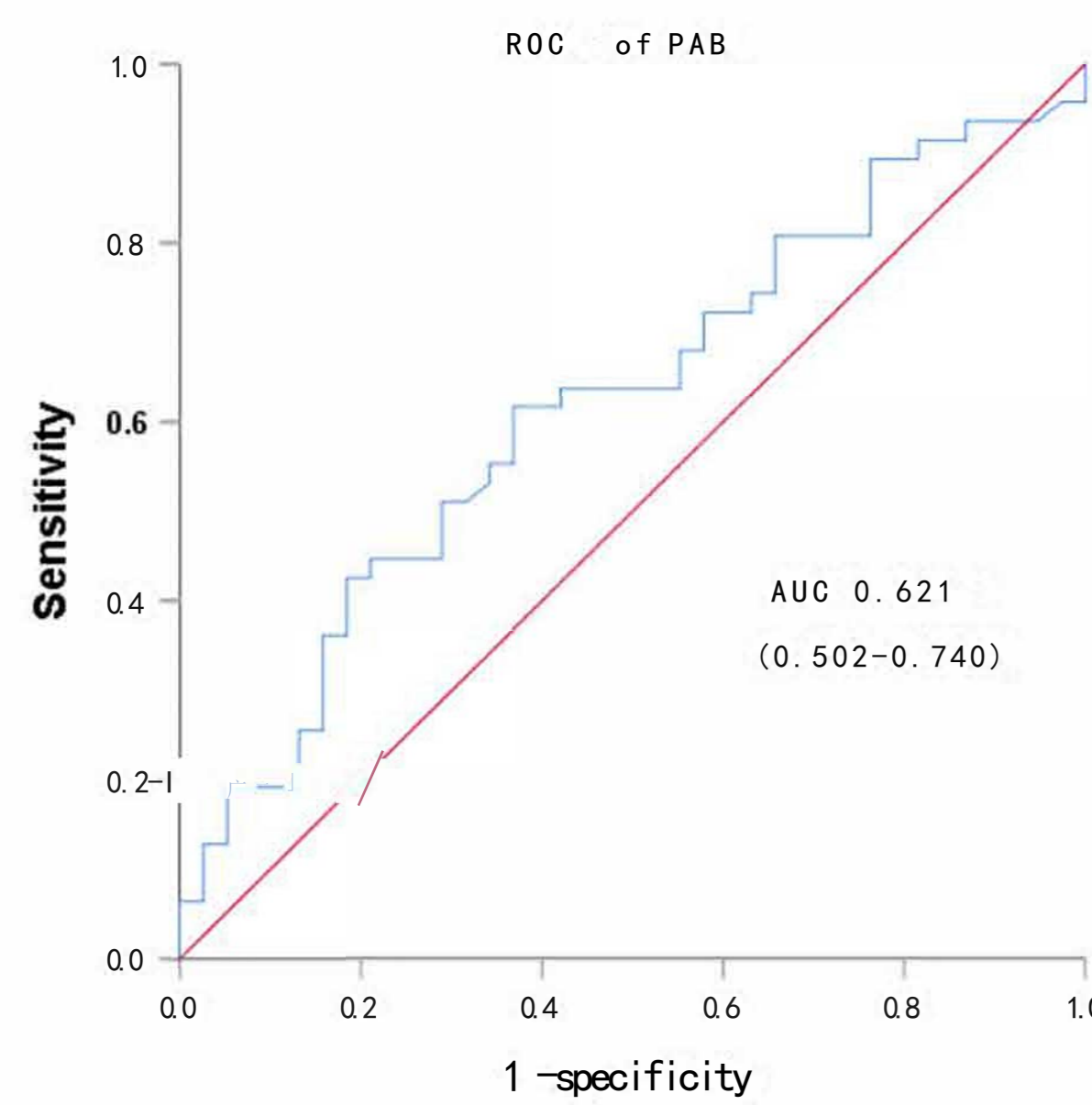

G

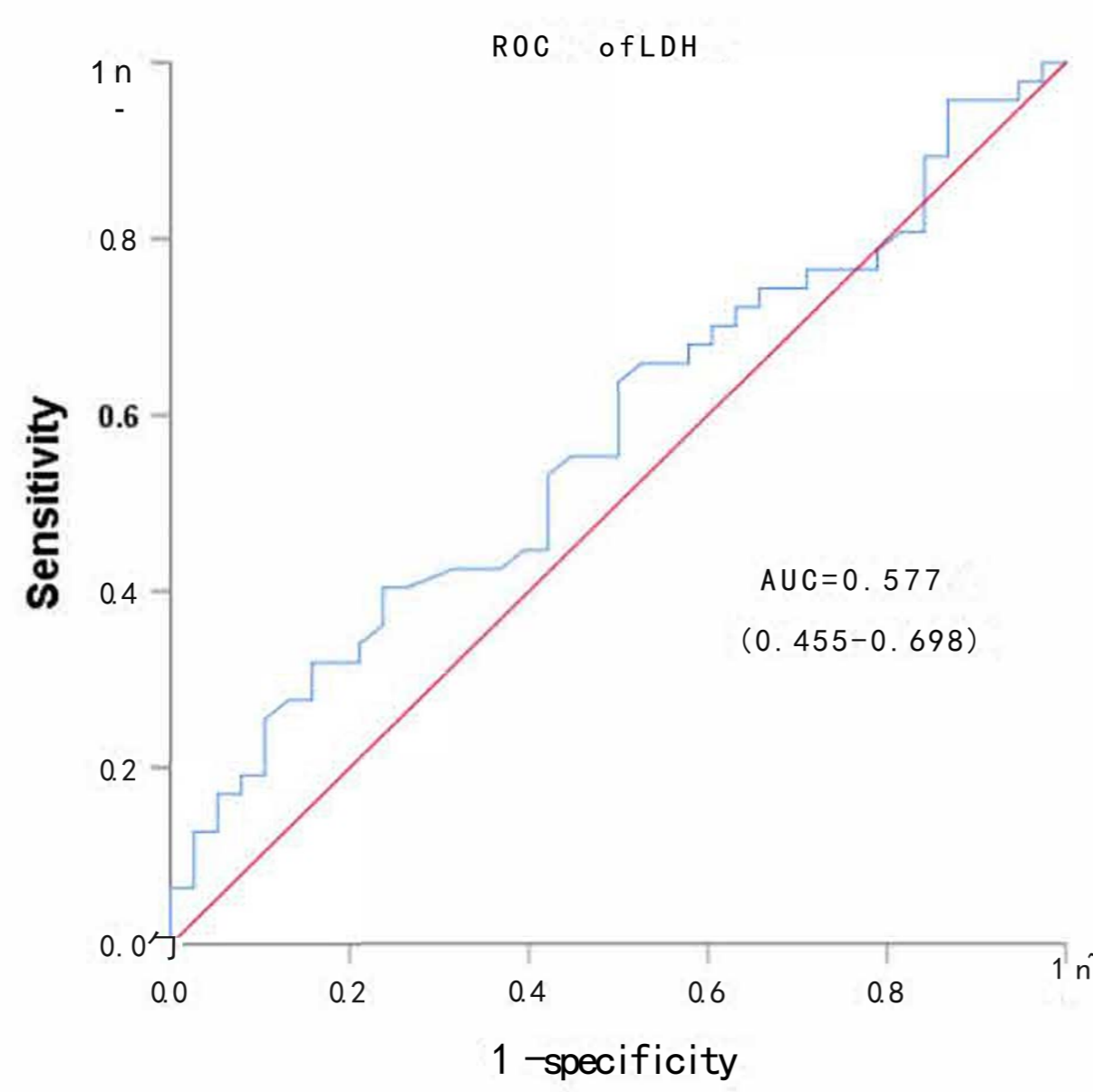

H

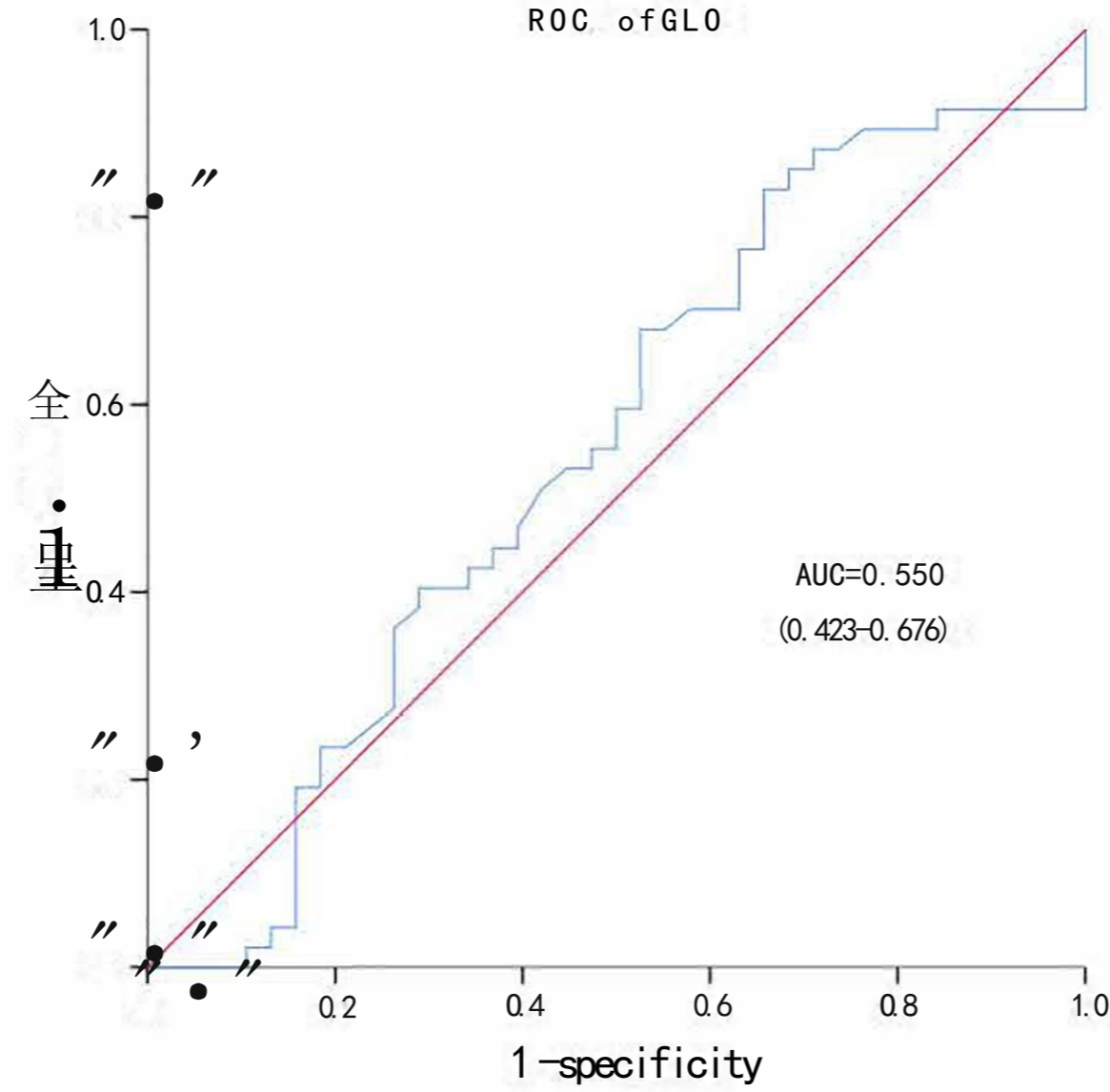

I

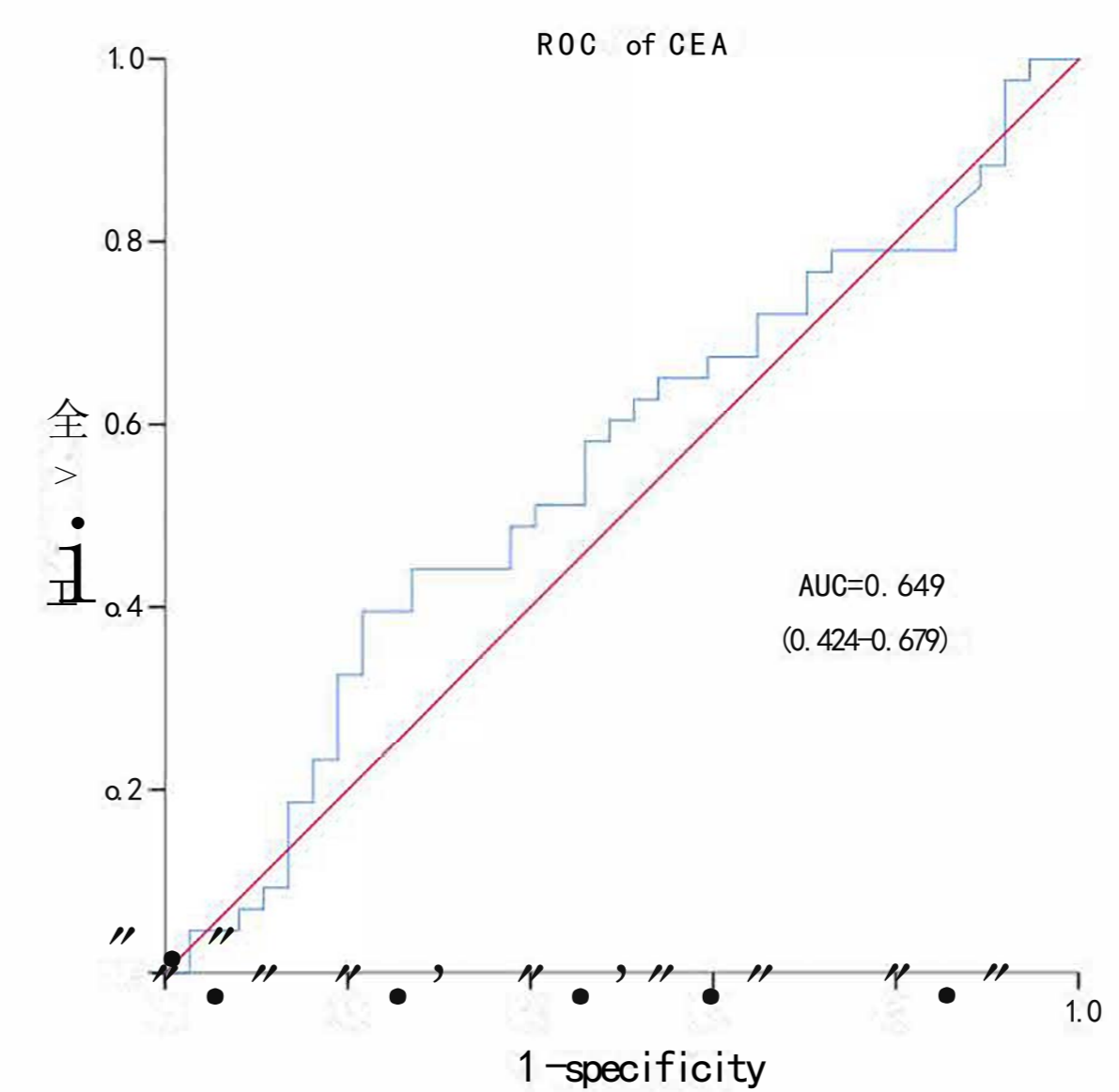

J

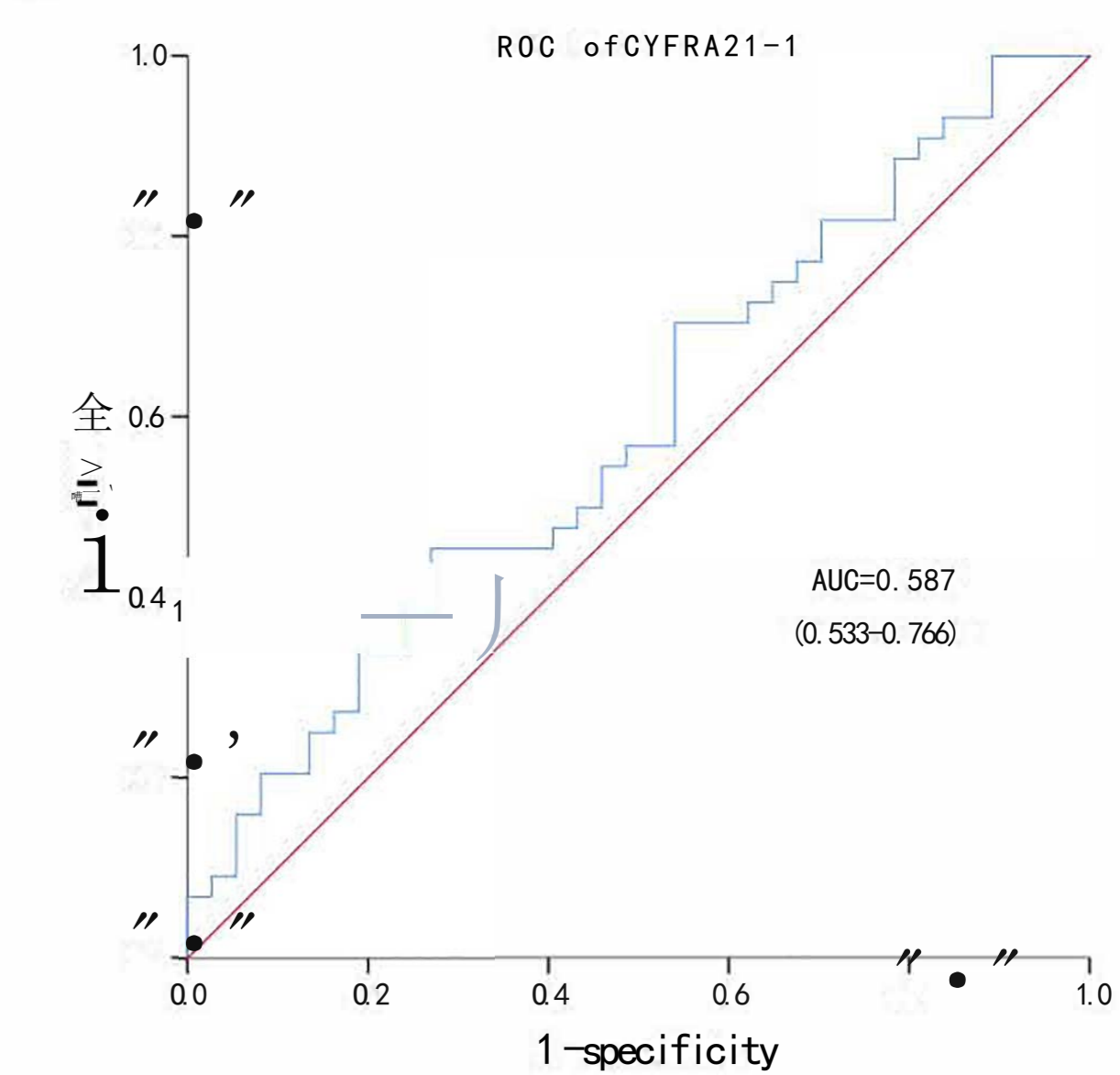

K

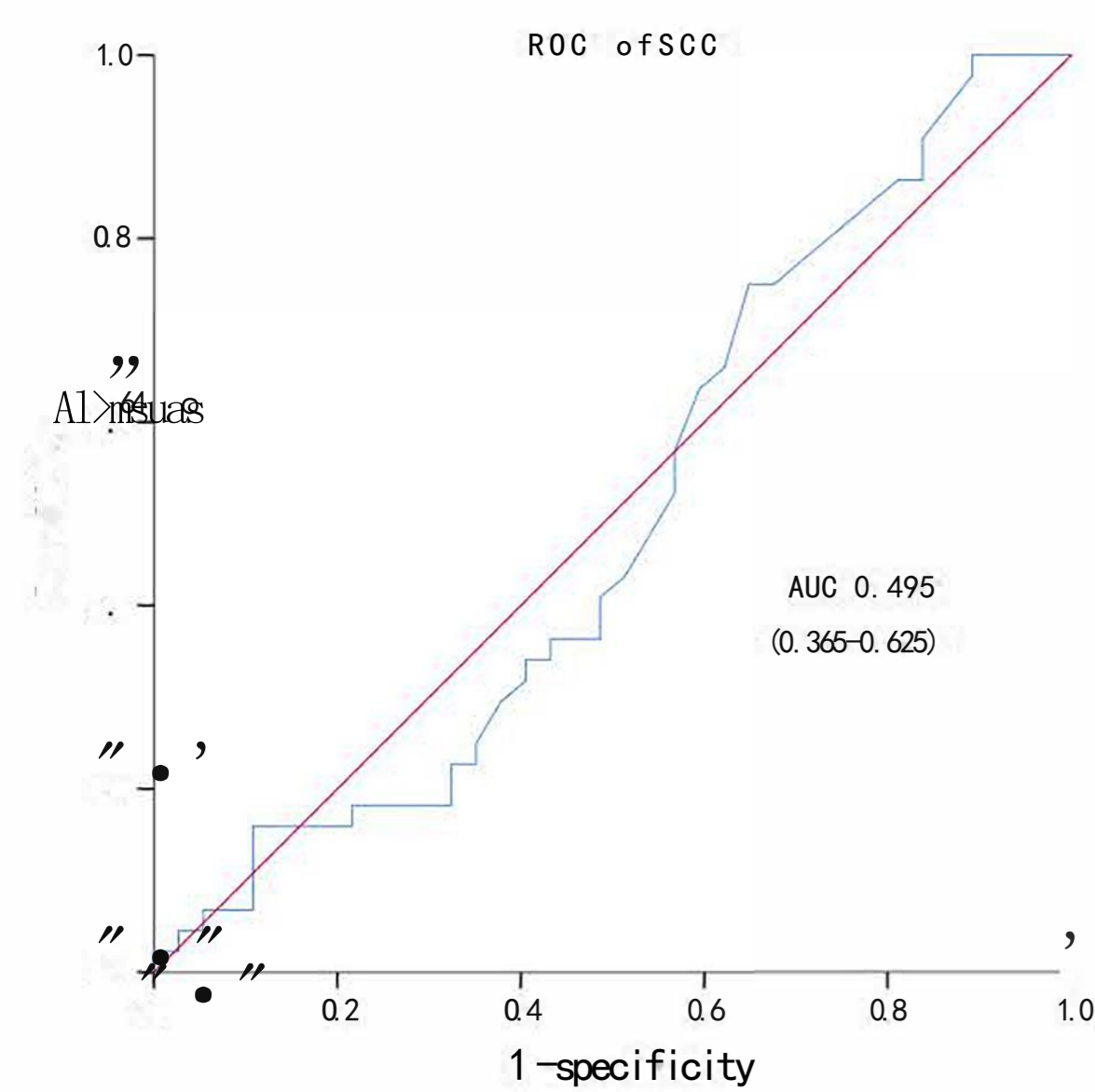

L

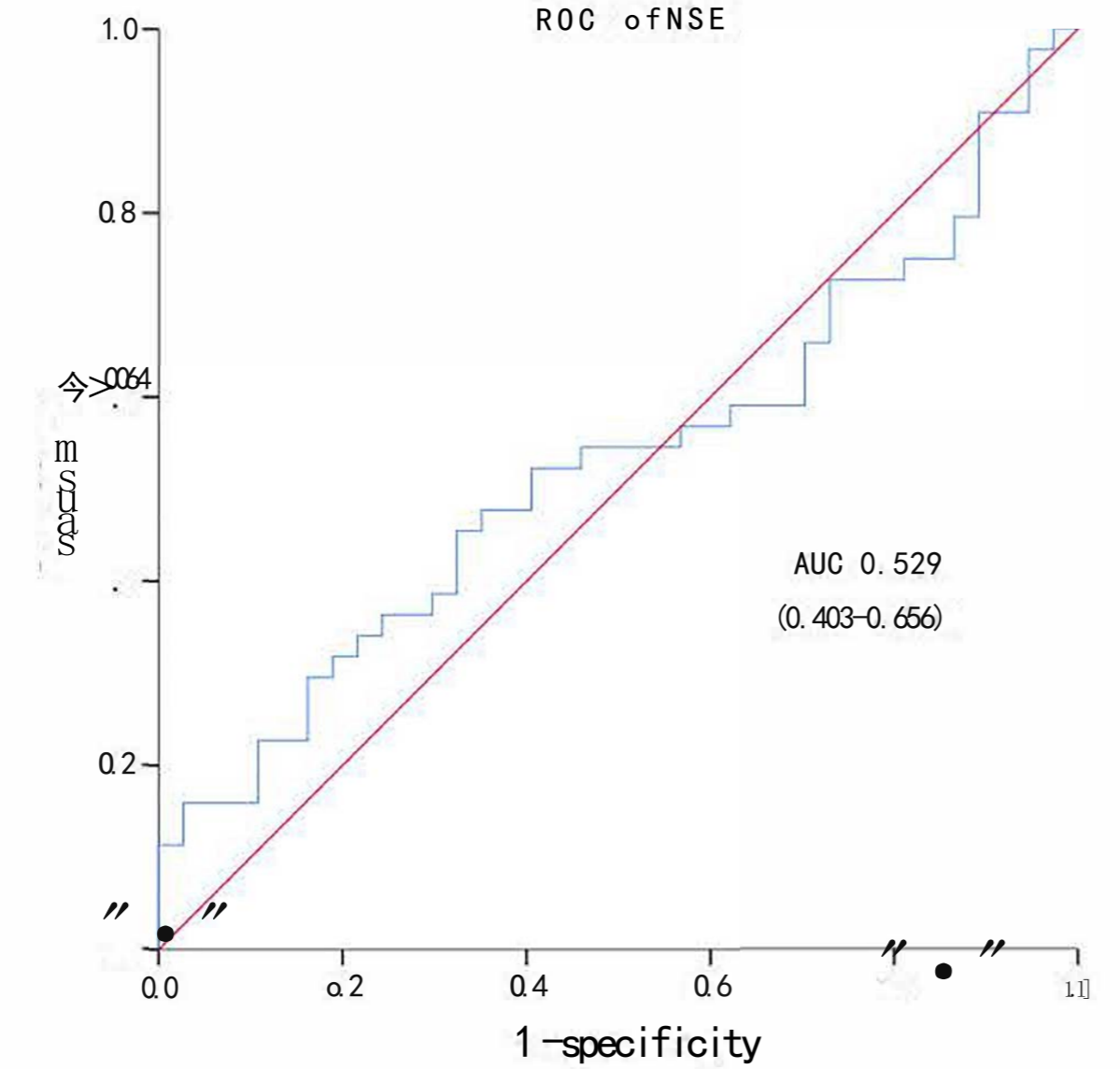

M

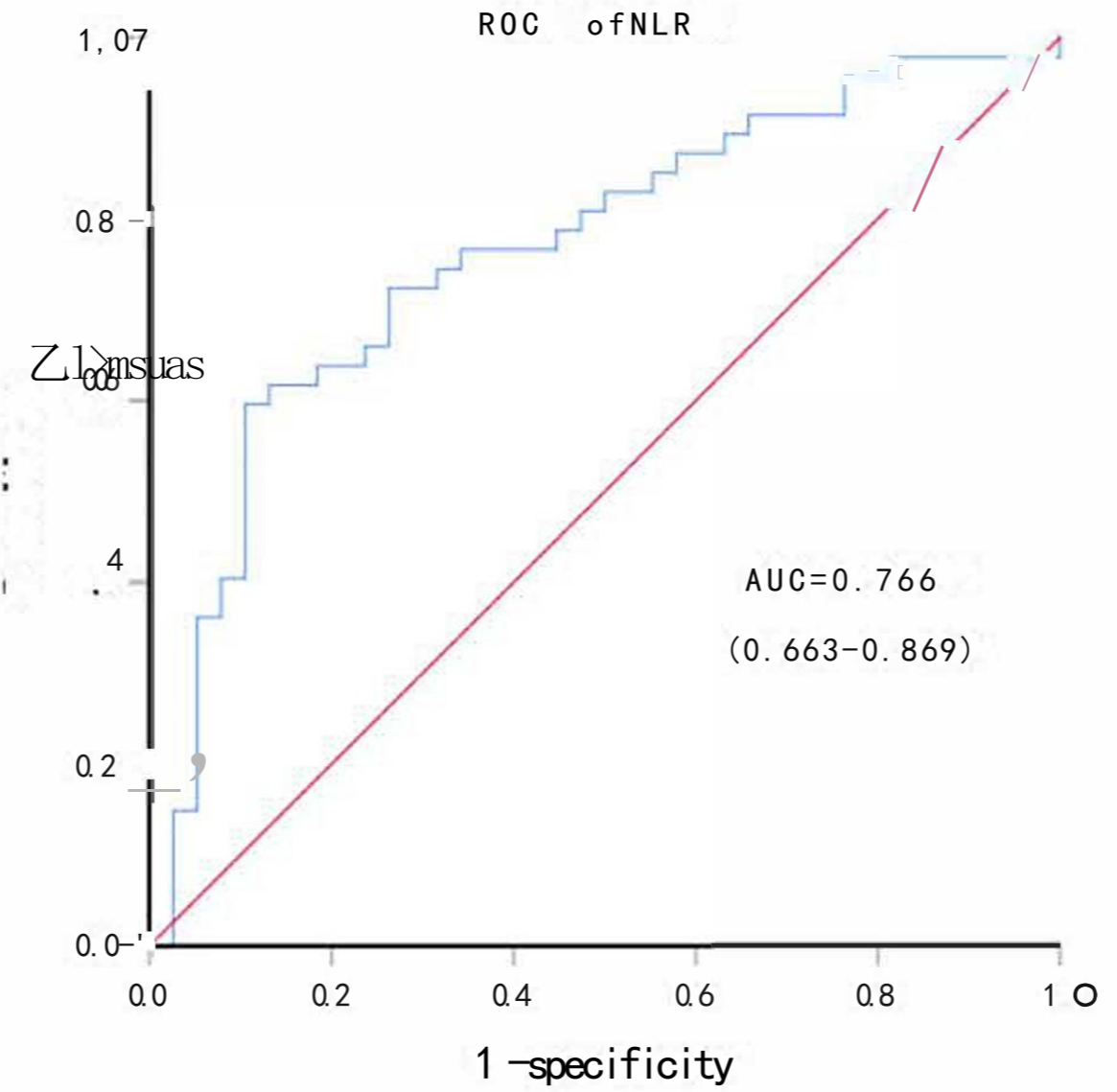

N

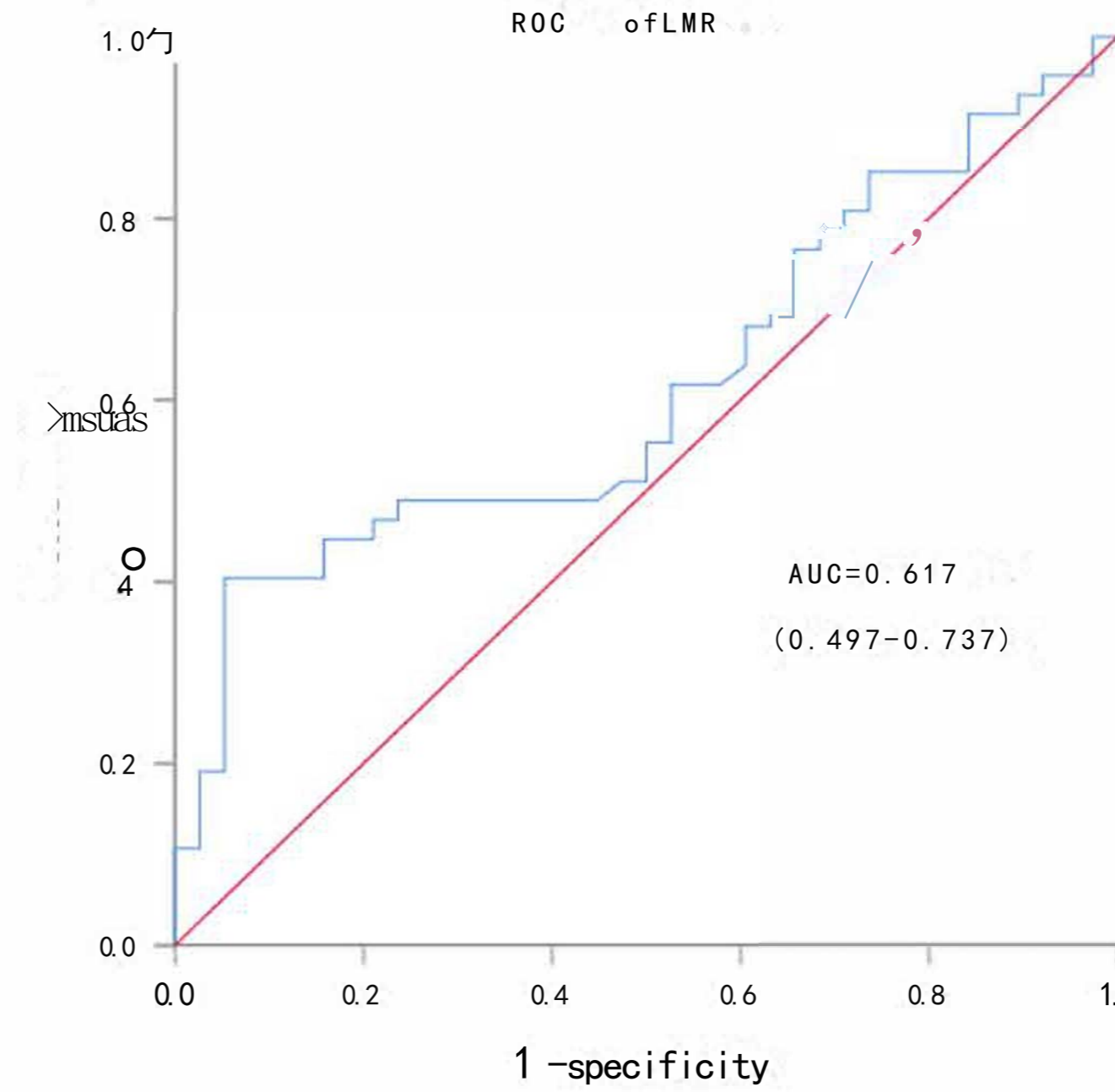

O

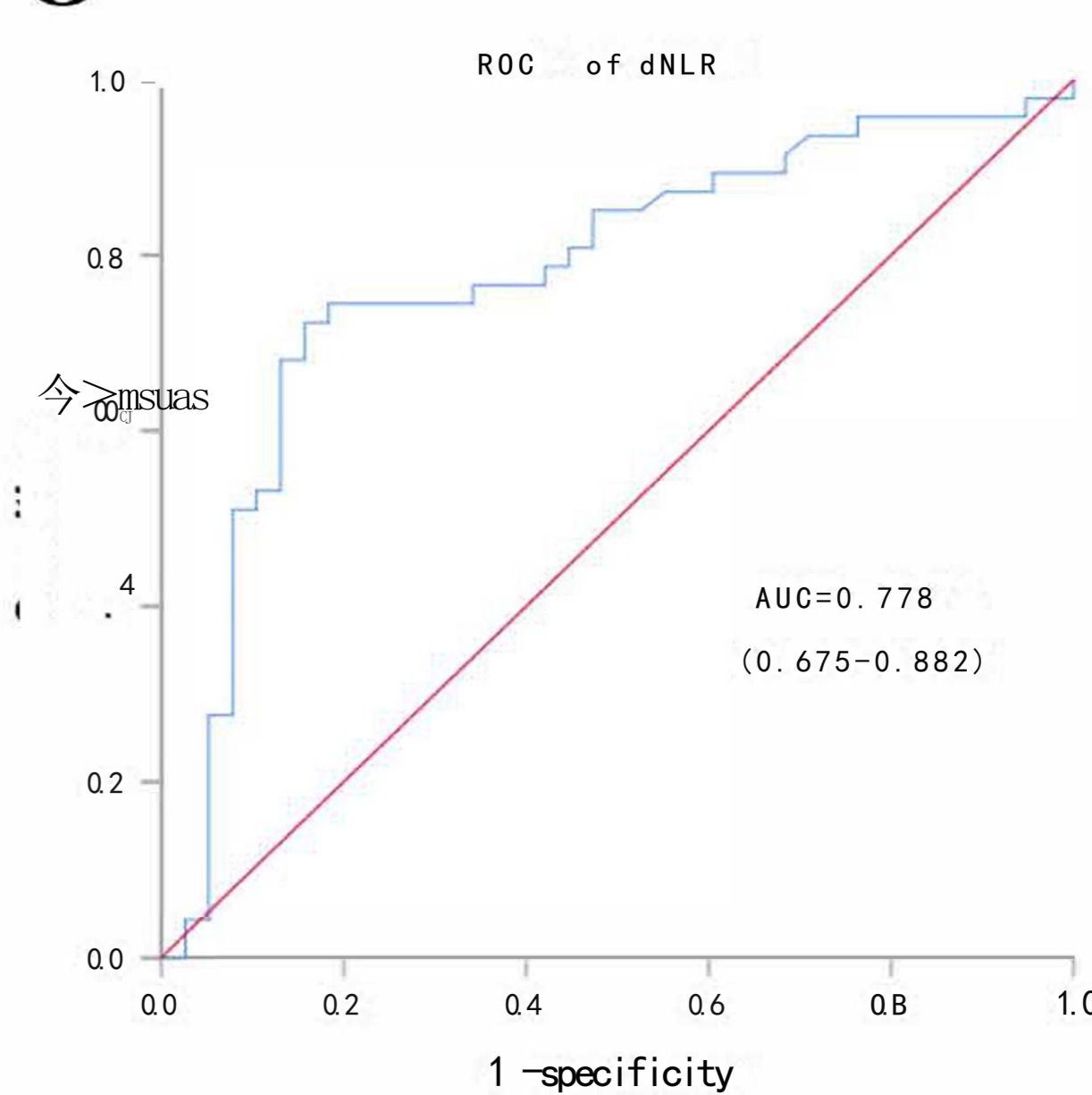

Supplement: Supplementary 2 — Figure S2: AUC and 95% CI of indicators. [file 7137357.f2.pdf]
